# Disruption of ataxia telangiectasia–mutated kinase enhances radiation therapy efficacy in spatially directed diffuse midline glioma models

**DOI:** 10.1172/JCI179395

**Published:** 2025-04-17

**Authors:** Avani Mangoli, Vennesa Valentine, Spencer M. Maingi, Sophie R. Wu, Harrison Q. Liu, Michael Aksu, Vaibhav Jain, Bronwen E. Foreman, Joshua A. Regal, Loren B. Weidenhammer, Connor E. Stewart, Maria E. Guerra Garcia, Emily Hocke, Karen Abramson, Tal Falick Michaeli, Nerissa T. Williams, Lixia Luo, Megan Romero, Katherine Deland, Samantha Gadd, Eita Uchida, Laura Attardi, Kouki Abe, Rintaro Hashizume, David M. Ashley, Oren J. Becher, David G. Kirsch, Simon G. Gregory, Zachary J. Reitman

**Affiliations:** 1 The Preston Robert Tisch Brain Tumor Center,; 2 Department of Radiation Oncology, and; 3 The Preston Robert Tisch Brain Tumor Center Omics Program, Duke University, Durham, North Carolina, USA.; 4 Department of Pediatric Hematology Oncology, Mount Sinai Kravis Children’s Hospital, New York, New York, USA.; 5 Department of Pediatrics, University of Alabama at Birmingham, Birmingham, Alabama, USA.; 6 Departments of Radiation Oncology and Genetics, Stanford University School of Medicine, Stanford, California, USA.; 7 Princess Margaret Cancer Centre, University of Toronto, Toronto, Ontario, Canada.

**Keywords:** Neuroscience, Oncology, Brain cancer, Drug therapy, Mouse models

## Abstract

Diffuse midline gliomas (DMGs) are lethal brain tumors characterized by p53-inactivating mutations and oncohistone H3.3K27M mutations that rewire the cellular response to genotoxic stress. We used RCAS/tv-a retroviruses and Cre recombinase to inactivate p53 and induce native H3.3K27M mutations in a lineage- and spatially directed manner. We generated primary mouse tumors that recapitulated human DMG. Disrupting ataxia-telangiectasia mutated (ATM) kinase enhanced the efficacy of radiation therapy (RT) in murine and patient-derived DMG models and increased survival. Microscopy-based in situ sequencing was used to spatially resolve transcriptional profiles in more than 750,000 single cells with or without ATM disruption and RT, revealing altered immune-neoplastic and endothelial cell interactions after treatment. An allelic series of primary murine DMG models with different p53 mutations confirmed that transactivation-independent p53 activity was a key mediator of radiosensitivity after ATM disruption. We generated primary DMG mouse models and performed deep profiling that revealed mechanisms of response to ATM disruption and RT that can be utilized as a therapeutic strategy.

## Abstract 2

Our work provides insight into mechanisms of resistance and biomarkers required to design future trials of ATM inhibitors in diffuse midline glioma patients.

## Graphical abstract

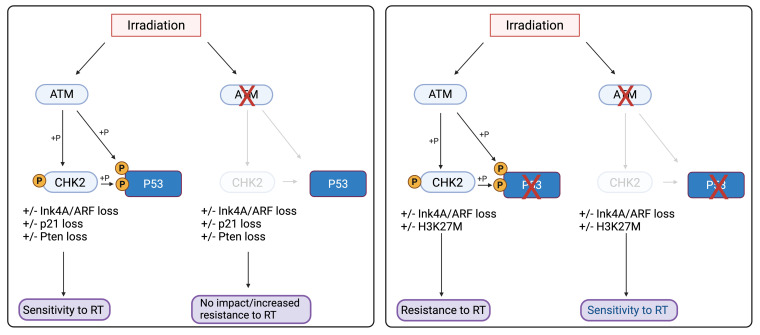

## Introduction

Diffuse midline gliomas (DMGs) are lethal brain tumors that occur in children and young adults. These tumors are localized in essential midline brain structures, such as the brainstem and thalamus, making them surgically inoperable and unresponsive to conventional chemotherapy. The median overall survival of patients with DMGs is less than 2 years. Although radiation therapy (RT) may improve symptoms and extend life, it remains palliative. Somatic activation of lysine 27 to methionine mutations in histone variant 3.3 (H3.3K27M) is a defining feature of DMG (1, 2). Approximately 70% of DMGs harbor inactivating mutations in the tumor suppressor *TP53* (1–3) that are associated with radioresistance in patients and preclinical models (4, 5).

A key limitation of current primary DMG preclinical models is the ability to induce K27M mutations in the native *H3f3a* locus in a spatially, lineage-, and temporally controlled manner. Patient-derived xenografts (6), patient-derived cell lines (7), in utero electroporation (7), and syngeneic mouse models (8, 9) have provided key insights into this disease. A conditional H3f3a-loxP-Stop-loxP-K27M-Tag allele (H3f3a^LSL-K27M-Tag^) has also been generated that allows the expression of H3.3K27M from the endogenous mouse *H3f3a* locus in the presence of Cre recombinase (10). However, this model has been limited by cell lineages that can be interrogated with existing Cre driver lines, such as *Nestin*-Cre (10). To date, the conditional H3.3K27M alleles have not been investigated in an entirely spatially controlled manner. We and others have used the RCAS/tv-a retroviral system for spatially directed modulation of glioma tumorigenesis in mice (5, 11–15). The RCAS/tv-a platform was used to deliver an exogenous H3.3K27M (13, 14, 16), but to our knowledge, it has not been used to edit the endogenous *H3f3a* allele. A variety of model systems have been used to investigate the mechanisms associated with the development of DMG and to assess therapeutic strategies.

Inhibition of ataxia-telangiectasia mutated (ATM) kinase has emerged as a strategy to enhance the efficacy of RT for DMG (17). ATM is a master orchestrator of the DNA damage response to double-stranded breaks (17). Patients with hereditary loss-of-function ATM variants and those with tumors containing ATM variants are extremely sensitive to RT (17). Consequently, a brain-penetrant ATM inhibitor has entered clinical trials for adult brain tumors (NCT03423628) (18). A recent study identified ATM inhibition as a potent radiosensitization strategy in various patient-derived pediatric high-grade glioma models (6). We found that functional ATM loss–radiosensitized primary mouse models of DMG were driven by p53 loss, but not WT p53 (5, 11). ATM loss increases tumor sensitivity to radiotherapy via radiosensitization of neoplastic cells rather than the vasculature (12). However, it remains uncertain whether H3.3K27M affects the ability of *Atm* loss to radiosensitize primary DMGs. This is of particular importance, since H3.3K27M regulates the p16 molecular checkpoint that regulates G_1_-to-S cell-cycle progression (13) and could thereby influence the radiation response.

Here, we examined strategies to exploit the genomically stressed cell state in H3.3K27M/TP53-altered DMG. We improved on previous models that delivered H3.3K27M from an exogenous RCAS payload (11, 13) by combining the RCAS/tv-a system with H3f3a^LSL-K27M-Tag^ mice to express H3.3K27M from the endogenous *H3f3a* locus. This autochthonous mouse model enabled us to analyze the effect of *Atm* loss in the context of H3.3K27M/TP53-altered brain tumors to mimic human DMG (10). We found that primary DMGs expressing H3.3K27M driven by p53 loss were radiosensitized by *Atm* loss. To explore the resistance mechanisms in specific tumor cells, we examined primary mouse DMGs after focal brain irradiation using high-resolution single-cell in situ sequencing (ISS). The results identified the overexpression of the cell-cycle regulator *Cdkn1a* as a putative resistance factor in *Atm*-intact DMG. We showed that *Cdkn1a*, or the transcriptional activity of p53 in general, was dispensable for DMG radiosensitization by *Atm* loss. Therefore, the nontransactivation functions of p53 may determine the sensitivity of DMGs to combinations of ATM inhibitors and RT. The high-resolution results describe a genetically faithful and flexible primary mouse model of DMG, identifying the mechanisms of resistance to a therapeutic strategy currently in clinical trials.

## Results

### Conditional p53 loss and H3.3K27M expression in retrovirus-induced mouse DMGs.

**Figure 1:**
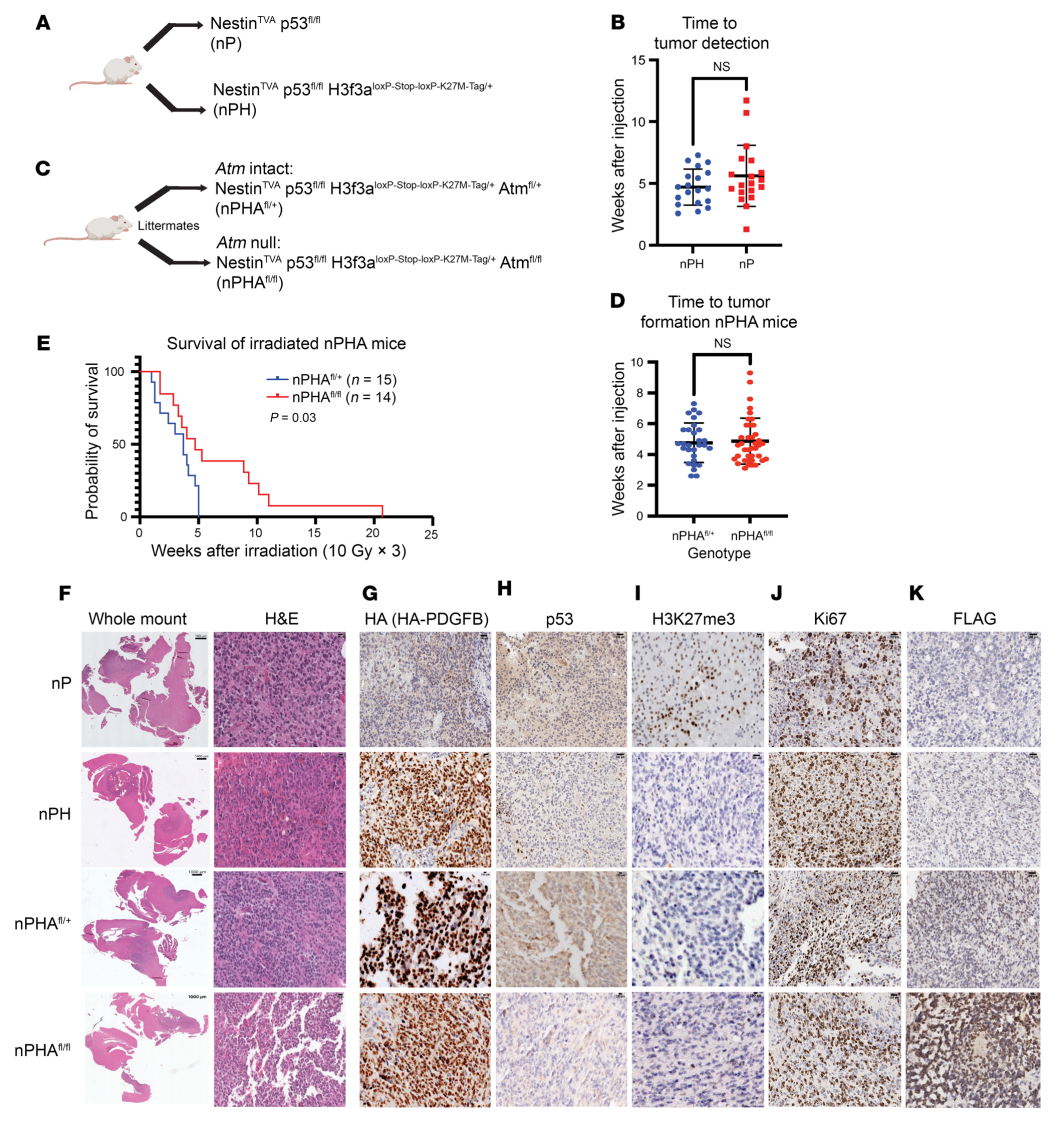
*Atm* loss improves radiosensitivity of primary murine DMBs generated using a conditional H3.3K27M allele. (**A**) Representation of mouse genotypes used to generate primary mouse DMGs with p53 loss (Nestin^TVA^ p53^fl/fl^ [nP]) and mouse DMGs with p53 loss and H3.3K27M (Nestin^TVA^ p53^fl/fl^ H3f3a^loxP-Stop-loxP-K27M-Tag/+^ [nPH]) with or without the conditional H3.3K27M allele. Mice also contained 1 intact and 1 floxed allele of *Atm* (Atm^fl/+^, not shown). (**B**) Dot plot showing the time to tumor formation between nPH and nP mice without any statistical significance. Welch’s *t* test was used to determine statistical significance. (**C**) Schematic showing nPHA^fl/+^ (Atm^fl/+^) and nPHA^fl/fl^ (Atm^fl/fl)^ within the RCAS/tv-a retrovirus and conditional H3K27M allele. **(D**) Time to tumor formation showing no statistical difference between nPHA^fl/fl^ and nPHA^fl/+^ mice. Welch’s *t* test was used to determine statistical significance. (**E**) Overall survival of nPHA^fl/fl^ and nPHA^fl/+^ mice following administration of 3 daily fractions of 10 Gy image-guided focal brain irradiation, with significantly longer median survival of nPHA^fl/fl^ mice. *P* = 0.03, by Mantel-Cox (log-rank) test. (**F**) Whole-mount and H&E images of tumor cells from nP, nPH, nPHA^fl/+^, and nPHA^fl/fl^ (top to bottom) mice exhibiting hypercellularity and infiltration of normal brain tissue. (**G**) IHC for HA expression indicating the presence of the PDGF-β HA tag. (**H**) IHC for p53. (**I**) IHC for histone 3 lysine 27 trimethylation (H3K27me3). (**J**) IHC displaying the Ki67 proliferation of tumor cells. (**K**) Anti-FLAG IHC confirmed the presence of the FLAG-HA tag. Scale bars: 100 μm (for all H&E and IHC images in **F**–**K**); 1,000 μm (for whole-mount images in **F**)

To express H3.3K27M from the endogenous *H3f3a* locus in retrovirus-induced primary mouse gliomas, we used a H3f3a^LSL-K27M-Tag^ allele that expresses H3.3K27M in the presence of Cre recombinase (10). To incorporate the H3f3a^LSL-K27M-Tag^ allele into the replication-competent avian sarcoma-leukosis virus (ASLV) long terminal repeat (LTR) with splice acceptor (RCAS/tv-a) retrovirus system, mice were bred with Nestin^TVA^ mice to allow RCAS retroviruses to specifically transduce TVA^+^ *Nestin–*expressing neural stem cells. To investigate the deletion of p53 specific to tumors, we crossbred a p53 variant in which critical exons were flanked by loxP sites (floxed), allowing for functional deletion of p53 in the presence of Cre recombinase. We first introduced retroviruses into Nestin^TVA^ p53^fl/fl^ H3f3a^LSL-K27M-Tag/+^ mice (hereafter referred to as nPH mice) and compared them with matched mice lacking the H3f3a^LSL-K27M-Tag^ allele (hereafter referred to as nP mice) (Figure 1A). We induced DMGs by injecting mice with RCAS retroviruses expressing Cre recombinase, firefly luciferase, and the oncogene platelet-derived growth factor–ligand β (*PDGFB*) and monitored them for tumor formation by in vivo imaging. Using luciferase-based bioluminescence imaging to detect tumors, we determined that there was no difference in time to tumor formation in H3f3a^LSL-K27M-Tag/+^ mice compared with matched mice lacking the H3f3a^LSL-K27M-Tag^ allele (Figure 1B). To investigate the effects of *Atm* deletion in these tumors, we also generated Nestin^TVA^ p53^fl/fl^ H3f3a^LSL-K27M-Tag/+^ Atm^fl/fl^ mice (hereafter referred to as nPHA^fl/fl^ mice) and littermate controls with intact *Atm* in their tumors of genotype Nestin^TVA^ p53^fl/fl^ H3f3a^LSL-K27M-Tag/+^ Atm^fl/+^ (hereafter referred to as nPHA^fl/+^ mice) (Figure 1, C–E, see description of *Atm* loss results below). Tumors exhibiting hypercellularity and diffuse infiltration of the nearby normal brain on H&E formed within 4–8 weeks with high penetrance (Figure 1F). We detected HA expression indicating the presence of the HA tag on both H3.3K27M and PDGF-β constructs (Figure 1G). As expected, p53 was not detected in p53^fl/fl^ tumors by IHC (Figure 1H). Histone 3 lysine 27 trimethylation was significantly decreased by IHC in H3f3a^LSL-K27M-Tag/+^ tumors compared with controls (mean, 50.49 % [nP] vs. 5.757 % [nPH] of cells staining positive, *P* < 0.001; Figure 1I and Supplemental Figure 1; supplemental material available online with this article; https://doi.org/10.1172/JCI179395DS1), indicating that H3.3K27M could functionally deplete H3K27me3 as predicted (19). Differentially methylated features between nP and K27M-bearing tumors showed hyper- and hypomethylated features within promoters (Supplemental Figure 2A) and enhancers (Supplemental Figure 2B) Additional analysis showed a difference in the percentage of methylation within hypomethylated tiles and de novo tiles in K27M tumors compared with nP and normal murine tissue (Supplemental Figure 2C and D). Differentially methylated genes yielded from the hypomethylated genomic regions were most enriched for processes involving neuronal development and differentiation, suggesting developmental properties for promoter and enhancer tiles (Supplemental Figure 2E). This finding is consistent with the DNA methylation state of other tissues of these specific tiles and with the role of K27M in regulating oncogenic and developmental processes (20). Sequence motif analysis identified differential methylation of motifs associated with the transcription factors Hoxd13 and Hoxa11 (Supplemental Figure 2F), which are known to be involved in hindbrain development. Ki67 was elevated in more than 50% of tumor cells regardless of H3.3K27M status (Figure 1J). Anti-FLAG IHC confirmed the presence of the FLAG tag on the H3.3K27M construct (Figure 1K). FLAG IHC demonstrated that H3.3K27M-Tag^+^ cells diffusely infiltrated from a hypercellular tumor core into the brain parenchyma, suggesting the diffuse, infiltrative biology seen in human DMG. These results demonstrate that RCAS/tv-a and a conditional H3f3a^LSL-K27M-Tag^ allele can be combined to target K27M to the *H3f3a* gene in time, lineage, and space to generate primary mouse DMGs that recapitulate human disease.

### Atm loss radiosensitizes primary p53-null/H3.3K27M DMGs.

Targeting ATM kinase has emerged as a potential strategy to increase the efficacy of standard-of-care RT for brain tumors (5, 6, 17). We sought to determine whether disruption of ATM could radiosensitize primary mouse DMGs with p53 and H3.3K27M alterations. Previously, we established that *H3f3a*-WT brainstem gliomas lacking *Atm* in tumor cells were radiosensitized compared with littermate controls with a functional *Atm* allele in their tumors (5). However, these mice lack H3.3K27M, which disrupts the G_1_-to-S cell-cycle checkpoint (13) and may thereby affect the downstream effects of ATM deficiency (17). We hypothesized that *Atm* inactivation in the presence of the H3.3K27M allele would also radiosensitize tumors. To test this, we assessed the tumor-free survival of nPHA^fl/fl^ mice and compared their survival rates with those of control mice with intact ATM in their tumors of genotype nPHA^fl/+^ (Figure 1C). We found no difference in tumor-free survival between nPHA^fl/fl^ and nPHA^fl/+^ mice in the absence of irradiation (Figure 1D). To test whether *Atm* deletion radiosensitizes p53-null/H3.3K27M DMGs, we delivered 3 daily fractions of 10 Gy focal brain irradiation to mice using the Small Animal Radiation Research Platform (SARRP). nPHA^fl/fl^ mice had significantly longer median survival than did nPHA^fl/+^ mice (*P* = 0.03, Mantel-Cox [log-rank test], Figure 1E). Thus, *Atm* deletion in tumor cells enhanced the efficacy of focal brain irradiation for primary p53-null/H3.3K27M DMGs. H&E staining confirmed tumor cell presence and infiltration (Figure 1F), followed by IHC confirmation of HA expression (Figure 1G), p53 loss (Figure 1H), and the presence of H3.3K27M (Figure 1I), Ki67 (Figure 1J), and anti-FLAG antibodies (Figure 1K). These results show that *Atm* disruption enhanced the efficacy of RT for primary mouse DMGs that had p53 loss and the H3.3K27M mutation.

### In situ multiplexed microscopy reveals cell-cycle and semaphorin pathway changes after irradiation and Atm disruption.

**Figure 2:**
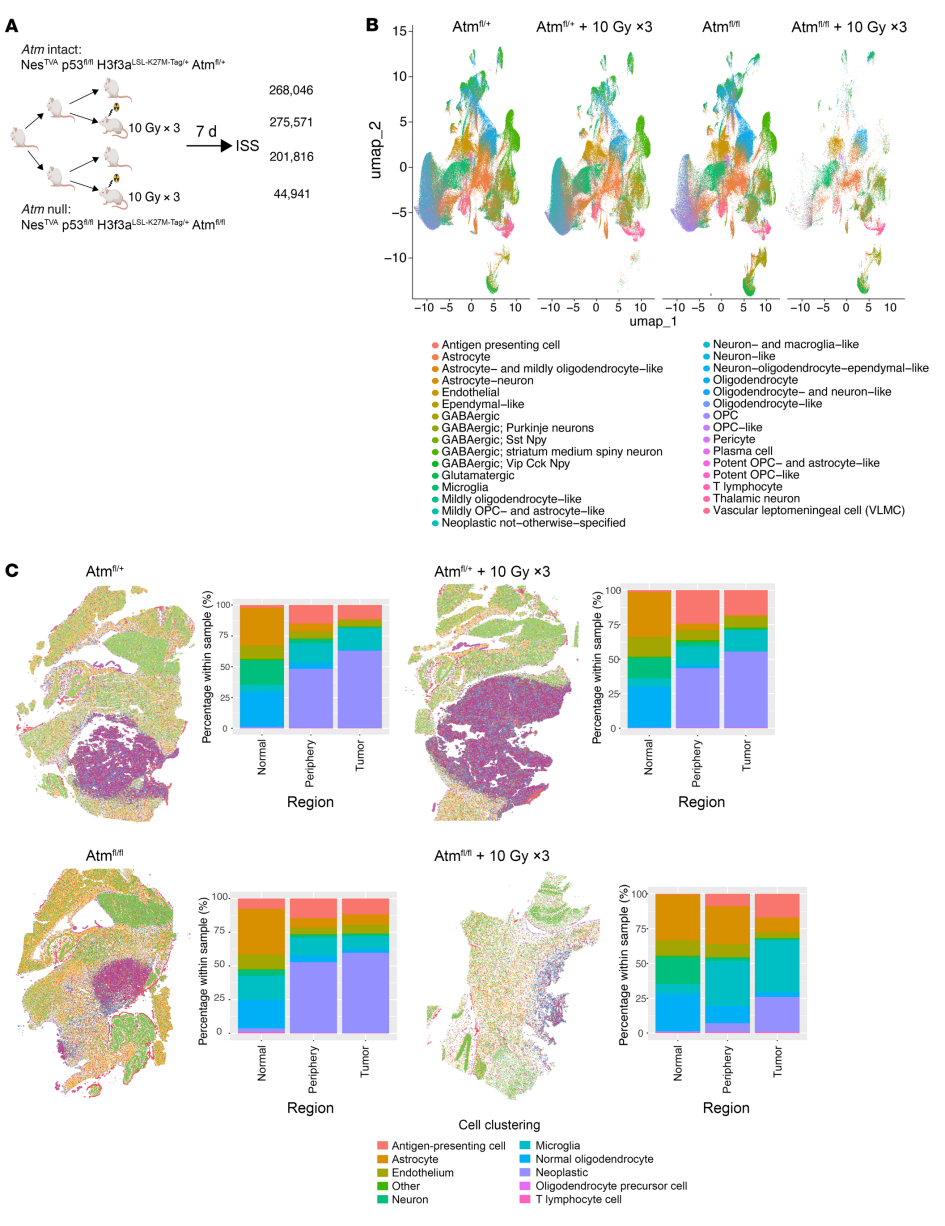
Spatial clustering in primary mouse DMGs treated with focal brain irradiation or tumoral *Atm* deletion. (**A**) Schematic of DMG-bearing mice were subjected or not to focal brain irradiation and ISS. All mice were of the genotype Nestin^TVA^ p53^fl/fl^ H3f3a^loxP-Stop-loxP-K27M-Tag/+^ with either *Atm-*intact (Atm^fl/+^) or *Atm-*null (Atm^fl/fl^) tumors. (**B**) Harmony integration showing clustering of 4 tumor-bearing mice with the H3f3a^loxP-Stop-loxP-K27M-Tag/+^ genotype with either *Atm-*intact (Atm^fl/+^) or *Atm-*null (Atm^fl/fl^) tumors. (**C**) Spatial clustering of cells into 10 cell archetypes based on label transfer in 4 tumor-bearing mouse brains (bottom color panel), H&E images of whole brain (left), and distribution of cells within normal brain, tumor periphery, and tumor core annotated in bar graph (right). Top row indicates Atm intact with and without irradiation. Bottom row indicates Atm null with and without irradiation. Color legend on the bottom corresponds to individual cell type noted on bar graph.

To explore the mechanisms underlying radiation efficacy and resistance, we performed spatially resolved gene expression analyses of primary mouse DMGs. Our previous work identified key differences in the response to irradiation and *Atm* loss between the neoplastic and vascular compartments within primary mouse tumors (12). To distinguish compartment-specific changes in gene expression, such as vascular and immune cells in specific regions of the tumor and nontumor brain, we needed to profile expression changes at single-cell resolution and in a spatially resolved manner. To achieve such a resolution, we used the 10x Genomics Xenium ISS platform to profile primary p53-null/H3.3K27M mouse DMGs. We examined DMG-bearing mice treated or not with focal brain irradiation (10 Gy × 3), with or without tumor *Atm* loss, as depicted in Figure 2A. We examined 5 μm mid-sagittal sections of formalin-fixed, paraffin-embedded (FFPE) tumor-bearing brains. We supplemented 10x Genomics’ standard mouse brain content with a custom panel containing padlock probes, resulting in 298 brain- and DMG-specific mRNA transcript assays (Supplemental Table 1). Individual cells were detected by nuclear DAPI staining, and cell boundaries were defined by in silico segmentation (see Methods). This yielded 790,374 individual cells across the 4 tumor-bearing brains. Next, we clustered cells on the basis of their transcriptional profiles and compared cell-type composition between the samples. Uniform manifold approximation and projection (UMAP) (21) was performed for reduction, projection, and harmony integration of differentiated normal and neoplastic brain cells into 20 and 29 clusters per specimen, respectively (Figure 2B and Supplemental Figure 3). Examination of differentially expressed marker genes in each cluster identified neoplastic and normal cells including GABAergic interneurons marked by *Gad1* and *Gad2*; microglia marked by *P2ry12*, *Lyz2*, and *C1qa*; and endothelial cells marked by *Cd34, Fn1*, and *Adgrl4* (Supplemental Figure 4). We used canonical cell-type markers and label transfer–based methods to collapse cell clusters into 10 cell archetypes (neoplastic, endothelial, neuronal, astrocytic, oligodendrocytic, microglial, T lymphocytic, etc.) that could be directly compared across specimens (Supplemental Table 2 and Supplemental Figure 5). This analysis revealed mass-like tumors with infiltrating edges recapitulating diffuse glioma biology (Figure 2C). Notably, an *Atm*-null post-irradiation tumor was smaller and involuted, which was suggestive of rapid treatment response. The tumor core, periphery, and nontumor areas were contoured using these data to allow comparisons between matching cell types and locations after irradiation or *Atm* loss (Figure 2C).

**Figure 3:**
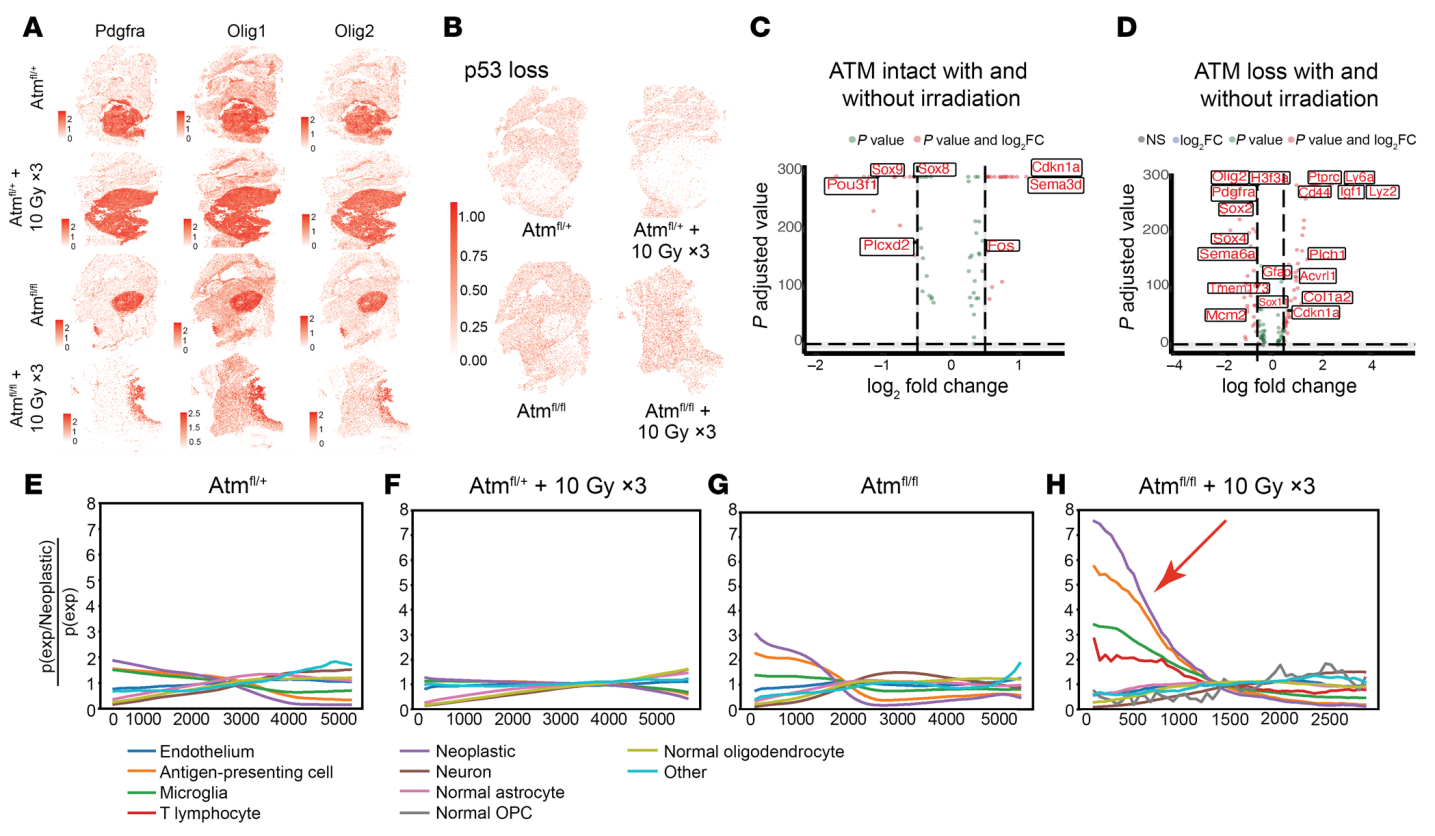
Differentially expressed genes and neighborhood analysis of primary mouse DMGs with tumoral *Atm* loss and/or focal irradiation. (**A**) Spatial identification of tumors by expression of *Pdgfra*, *Olig1*, and *Olig2* in all conditions (top to bottom): *Atm*-intact, *Atm*-intact with irradiation, *Atm*-null, *Atm*-null with irradiation. (**B**) Spatial identification of p53 loss in all tumor conditions: *Atm*-intact without and with irradiation (top row, left to right). *Atm*-null without and with irradiation (bottom row, left to right). (**C**) Key differentially expressed genes in *Atm*-intact neoplastic tumor cells treated with and without focal brain irradiation. The log_2_ fold change and *P* values for all genes are indicated in Supplemental Table 3. (**D**) Key differentially expressed genes in *Atm*-null neoplastic tumor cells treated with and without focal brain irradiation. The log_2_ fold change and *P* value for all genes are indicated in Supplemental Table 4. (**E**) Co-occurrence plot of *Atm*-intact (nPHA^fl/+^) tumor showing the number compared with the distance of various cell types in relation to neoplastic cells. (**F**) Co-occurrence plot of *Atm*-intact (nPHA^fl/+^) tumor with irradiation showing the number compared with the distance of various cell types in relation to neoplastic cells. (**G**) Co-occurrence plot of *Atm*-null (nPHA^fl/fl^) tumor showing the number compared with the distance of various cell types in relation to neoplastic cells. (**H**) Co-occurrence plot of *Atm*-null (nPHA^fl/fl^) tumor with irradiation showing the number compared with the distance of various cell types in relation to neoplastic cells. Red arrow indicates increased frequency of immune cells compared with neoplastic cells. Color legend for **E**–**H** is on the right side panel. OPC, oligodendrocyte precursor cell; TLC, T lymphocyte. Neighborhood enrichment and co-occurrence analyses were conducted on the entire slide. All unlabeled cells were removed for analysis.

We used the spatially resolved expression data to identify differentially expressed genes among neoplastic cells within the tumor cores. We first localized the tumor core within the full-brain sagittal sections using the canonical DMG neoplastic cell markers *Olig1*, *Olig2*, and *Pdgfra* (Figure 3A). As expected, we could not detect *p53* in the neoplastic cells within the tumor core in the Tp53^fl/fl^ model, whereas low baseline levels could be detected in non-neoplastic cell types (Figure 3B). Similarly, *Atm* transcripts were nearly undetectable in neoplastic cells from *Atm*-null tumors (mean fold-change –0.636, *P* < 0.0001 vs. *Atm*-intact tumor; Supplemental Figure 6). To identify transcripts that may be differentially expressed after irradiation and/or *Atm* loss, we interrogated differentially expressed genes in neoplastic cells after focal brain irradiation in *Atm* intact tumors (Supplemental Table 3) and *Atm*-null tumors (Supplemental Table 4). Cyclin-dependent kinase 1a (*Cdkn1a*), which encodes p21, a potent regulator of cell-cycle progression at G_1_, was the most differentially expressed gene after focal brain irradiation among *Atm* intact tumors (log-fold change 0.8, *P* = 0.0001, by Wilcoxon test, Figure 3C). *Cdkn1a* was still upregulated, albeit to a lesser degree, after focal brain irradiation among *Atm*-null tumors (log-fold change 0.6, *P* = 5.46 × 10^–8^, by Wilcoxon test, Figure 3D). Conversely, transcription factors such as *Sox8* and *Sox9*, which are associated with developmental cell states, were substantially downregulated after irradiation in *Atm*-intact tumors, whereas *Sox2*, *Sox4*, *Pdgfra*, and *Olig2* were associated with early glial differentiation were all substantially downregulated after irradiation in *Atm*-null tumors. These results identify the differential expression of cell-cycle regulators and cell-fate–regulating transcription factors after irradiation in a primary DMG mouse model.

Irradiation and *Atm* loss were associated with changes in the expression of semaphorin genes specifically, semaphorin 6A (*Sema6a*) and semaphorin 3D (*Sema3d*) which have been implicated in the tumor cell proliferation and survival in glioma mouse models and in glioblastomas (22, 23). After irradiation in *Atm*-intact tumors, *Sema3d* was significantly increased (log-fold change 1.13, *P* = 0), suggesting that RT may influence proliferation within the neoplastic core. After irradiation of *Atm*-null tumors, *Sema6a* was significantly decreased (log-fold change –0.40, *P* = 6.59 × 10^–15^). We utilized single-nucleus RNA-Seq (snRNA-Seq) data from additional primary murine models derived via in utero electroporation approaches to validate semaphorin, p21, and endothelial cell interactions in orthogonal models (Supplemental Figure 7) (9). However, our single-cell spatial transcriptomics provided additional mechanistic insight indicating that after radiotherapy, specific semaphorin genes are altered in neoplastic cells that might play a critical role in glioma biology.

### Neighborhood analysis shows altered immune-neoplastic interactions after treatment.

Targeting ATM combined with irradiation can bridge innate and adaptive immune processes in extracranial cancers (24, 25). This led us to interrogate the spatial relationship between neoplastic cells and the immune microenvironment. We examined whether the proximity between neoplastic cells and normal cells varied across irradiated or *Atm*-null tumors. Neighborhood analysis quantified the spatial proximity between different cell types and was used to estimate the mean distance between the neoplastic cells and other cell types (Supplemental Figure 8). These data identified an increased proximity of neoplastic cells and immune cells, such as antigen-presenting cells (APCs) and microglia, after *Atm* loss and after treatment with irradiation, which was especially pronounced in the irradiated *Atm*-null tumor. Colocalization analysis between neoplastic cells and other cell types confirmed that microglia and APCs were most enriched within 0–500 μm (Figure 3, E–H), and these cell types were most colocalized in the irradiated *Atm-*null tumor (Figure 3H).

### Ligand-receptor analysis reveals endothelial cell communication.

Next, cell-cell and cell-ligand-receptor interactions in primary mouse DMGs established that endothelial cells had the highest frequency of interactions (Supplemental Figure 9 and Supplemental Table 5). We evaluated statistically significant ligand-receptor interactions (*P* < 0.05) among the tumors and identified the interaction between the endothelium, microglia, and neoplastic cells with decreased Col1a2-CD93 receptor interaction after *Atm* loss and irradiation (Supplemental Figure 9). CD93 plays a role in tumor-associated vasculature (26), and changes in Col1a2 expression have been observed after radiotherapy in other cancers (27). These results provide insight into the changes in endothelial cell interactions after tumor irradiation. After irradiation of *Atm*-intact tumors, the cell-ligand interaction of Sema3a:NRP2 between neoplastic cells and microglia decreased. This interaction has been noted to affect glioma cell migration (28), implying a potential alteration in migration with irradiation. We observed the opposite effect in *Atm*-null tumors after irradiation (Supplemental Figure 9). Thus, ligand-receptor analysis of ISS data suggests that glioma-linked collagen and semaphorin interactions can be examined in primary DMG mouse models.

### Pharmacologic ATM inhibition deregulates DNA damage responses and improves survival.

**Figure 4:**
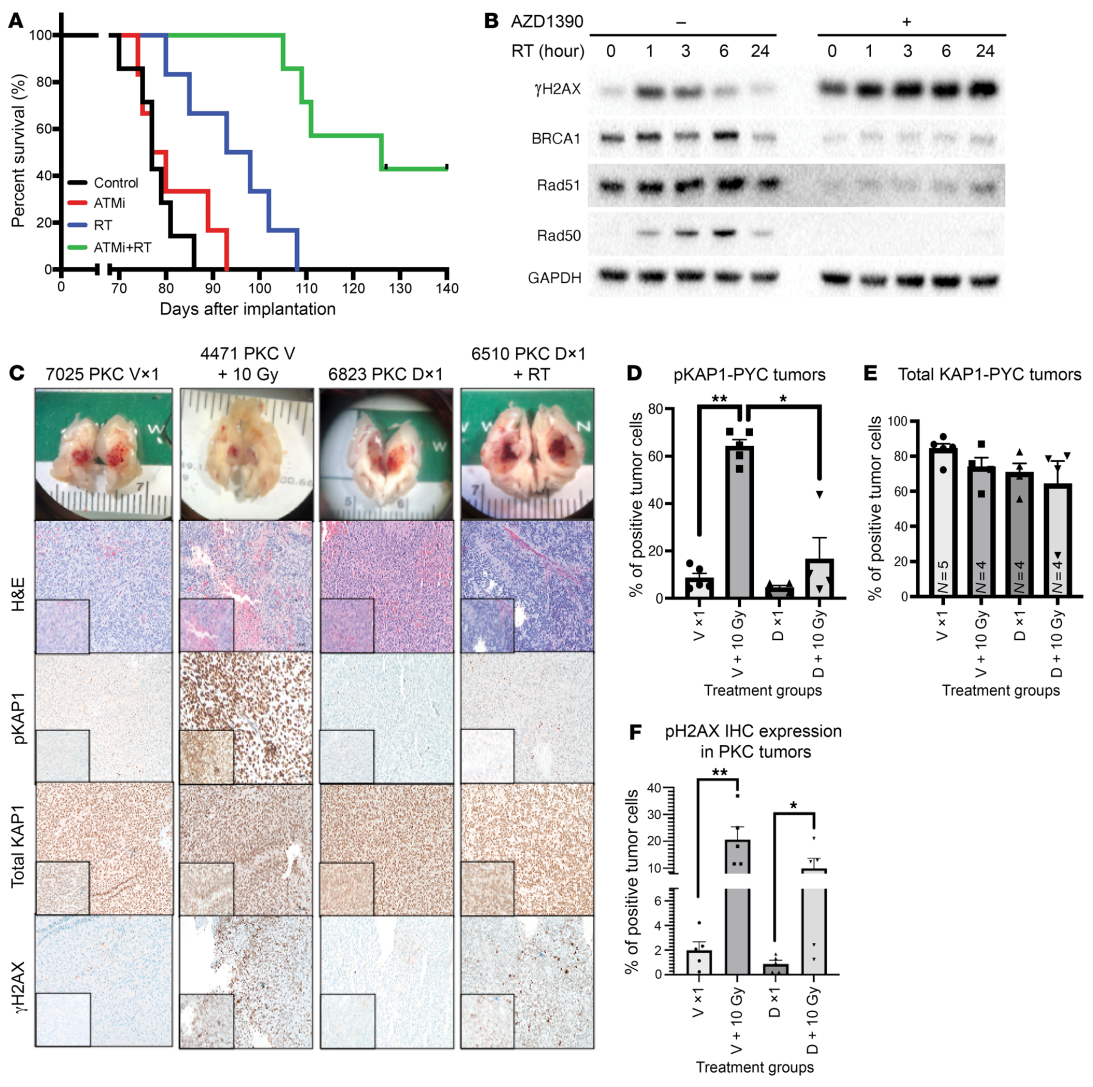
Pharmacologic inhibition and DNA damage response signaling in primary mouse DMGs with tumoral *Atm* loss and/or focal irradiation. (**A**) Overall survival of mice bearing patient-derived SF8628 DMG xenografts were treated with 20 mg/kg AZD1390 for 2 weeks (ATM inhibitor [ATMi], 5 days per week for 2 weeks) and/or focal brain irradiation (RT, 2 Gy for 3 days per week for a total dose of 12 Gy). (**B**) Western blot of SF8628 followed by AZD1390 treatment with and without RT (0 hours, 1 hour, 3 hours, 6 hours, 24 hours). (**C**) Gross dissection and images of the brain with tumor were captured. Representative IHC images of tumor cells from Nestin^TVA^ p53^fl/fl^ PDGF-β + H3.3K27M + Cre (PKC) mice treated with vehicle (V) or the ATM inhibitor drug (D) AZD1390, with or without 10 Gy irradiation, for the following (top to bottom): H&E, p-KAP1, total KAP1, and γH2AX. Imaged with Zeiss Axio imager. Original magnification, ×20, insets ×40. (**D**) PDGF-β + H3.3K27M + p53^fl/fl^ (*n* = 5 per treatment group) cells stained for p-KAP1 revealed increased p-KAP1 expression in samples treated with 1 dose of 10 Gy and showed significance in vehicle-treated samples (PKC + V +/– RT). *P* = 0.0079, by Mann-Whitney *U* test. This expression was significantly reduced in drug-treated tumors subjected to RT compared with vehicle-treated tumors subjected to RT, suggesting the ATM inhibitor AZD1390 sensitized the DIPG tumor–bearing mice to irradiation. *P = 0.0159 and **P = 0.0079, by Mann-Whitney *U* test. (**E**) PDGF-β + H3.3K27M + p53^fl/fl^ tumor cells stained for total KAP1 show unchanged levels across all treatment groups (*n* = 5 per treatment group). (**F**) PDGF-β + H3.3K27M + p53^fl/fl^ tumor cells stained with yH2AX demonstrated increased expression in samples treated with 1 dose of 10 Gy RT when compared with their respective non-RT-treated samples (*n* = 5 per treatment group). **P = 0.0079, by Mann-Whitney *U* test for the vehicle-treated groups and *P = 0.0317, by Mann-Whitney *U* test for drug-treated groups.

To validate these findings, we confirmed that pharmacological inhibition of ATM could radiosensitize patient-derived models of DMG. To do so, we tested whether the brain-penetrant ATM inhibitor AZD1390 (18), combined with focal brain irradiation, could similarly improve the survival of a patient-derived xenograft model of the H3.3K27M-mutant and p53-mutant diffuse midline glioma SF8628 (29–32), which lacks a functional ATM mutation (Supplemental Figure 10 and Supplemental Table 6). The combination of AZD1390 and irradiation significantly extended the median survival of mice compared with either treatment alone (Figure 4A). We tested an *Atm*-intact genetically engineered model with a combination of AZD1930 and irradiation, which led to a trend for extended median survival compared with irradiation alone (median 29 days vs. 10 days, *P* = 0.1, log-rank test; Supplemental Figure 11). These results confirmed that pharmacologic or genetic targeting of ATM can radiosensitize multiple types of in vivo DMG models.

We investigated the DNA damage response in the SF8628 line by performing Western blotting in cells treated with AZD1390 with or without irradiation, which showed increased expression of GH2AX up to 24 hours after irradiation (Figure 4B and Supplemental Figure 12). To further interrogate the effects of irradiation after treatment with AZD1390, we treated Nestin-Tva Cre p53^fl/fl^ PDGF-β H3.3K27M mice (13) with vehicle or drug, along with 10 Gy irradiation and harvested mouse brain tumors 1 hour after irradiation. AZD1390 effectively inhibited ATM, as indicated by significantly reduced phosphorylated KAP1 (p-KAP1) expression in the treated group when compared with expression levels in the control group (*P* = 0.01, Mann-Whitney *U* test) and increased GH2AX expression (*P* = 0.03, Mann-Whitney *U* test) in tumor-bearing mice (Figure 4, C–F). The differential change in GH2AX expression in tumor-bearing mice treated with AZD1390 when compared with RT alone indicated a synergistic effect.

### Atm radiosensitizes Cdkn1a-null primary murine DMGs.

**Figure 5:**
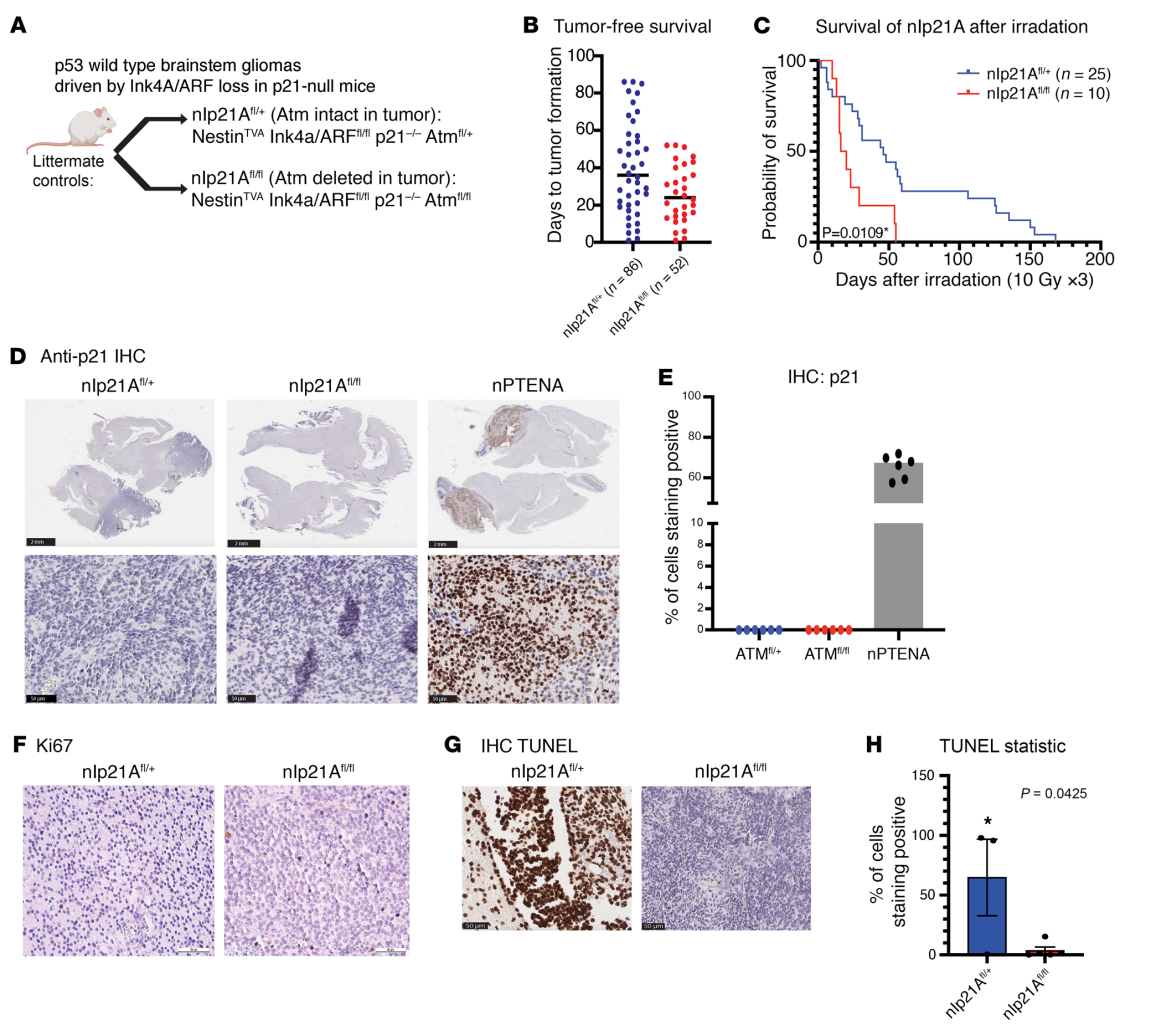
Effect of tumor-specific *Atm* loss in primary DMGs on a *Cdkn1a*-null (p21^–/–^)background. (**A**) Overview of the p21^–/–^ genotypes analyzed. (**B**) Tumor-free survival of nIp21A mice with and without intact *Atm* using the log-rank test. (**C**) Post-focal brain irradiation survival of nIp21A mice with and without intact *Atm* indicating a statistically significant survival benefit in nlp21A^fl/+^ mice (*P* < 0.05, by log-rank test). (**D**) IHC showing p21 expression in nIp21A mouse brains. Nestin^TVA^ Pten^fl/fl^ Atm^fl/+^ (nPtenA) tumor–bearing brain generated with identical RCAS viruses shown as a control. (**E**) Plot indicating the percentage of tumor cells that stained positive for p21 compared with the total cell count. (**F**) IHC images of staining for Ki67 showing proliferation for nlp21A^fl/+^ and nIp21A^fl/fl^. (**G**) TUNEL staining of tumor-bearing brains of nIp21A mice with and without intact *Atm* in tumors collected 1 hour after focal brain RT. (**H**) Quantification of TUNEL staining in nlp21A^fl/+^ mice. *P < 0.05, by unpaired *t* test. Scale bars: 2 mm (**D** top row) 50 μm, (**D** bottom row, **F**, and **G**).

Next, we dissected the specific functions of p53 that may affect the radiosensitivity of mouse DMG. Our primary models of DMG indicated that the presence of functional p53 was a key determinant of whether tumors were radiosensitized by *Atm* loss. For instance, primary p53-null/H3.3K27M tumors and primary p53-null/*H3f3a*-WT tumors were radiosensitized by *Atm* loss (Figure 1E and ref. 5, respectively). Conversely, p53 WT primary DMG models driven by inhibitor of cyclin-dependent kinase 4a (Ink4A) and alternative reading frame (ARF) or phosphatase and tensin homolog (PTEN) loss were not radiosensitized by *Atm* loss (5, 11). However, it is unknown whether the loss of p53 transcriptional activation and/or loss of other p53 functions enables radiosensitization by *Atm* loss. Our ISS data identified increased *Cdkn1a* expression in neoplastic cells after radiation. Since *Cdkn1a* (encoding p21) is a major transcriptional target of p53 (33), we hypothesized that the loss of *Cdkn1a* function downstream of p53 may be a key determinant of whether *Atm* loss can radiosensitize primary DMGs. To test whether *Cdkn1a* loss allows primary mouse DMGs to be radiosensitized by *Atm* loss, we examined our model of WT p53 DMGs driven by Ink4A/ARF loss, which was not radiosensitized by *Atm* loss (Nestin^TVA^ Ink4A/ARF^fl/fl^) (5). To test whether p21 loss could radiosensitize these mice when *Atm* was lost, we bred mice with constitutive p21 loss into this genotype (Nestin^TVA^ p21^–/–^ Ink4A/ARF^fl/fl^ Atm^fl/fl^, referred to hereafter as nlp21A^fl/fl^ mice). We tested the effects of tumor-specific *Atm* loss by comparing these mice with their littermate controls with intact *Atm* with the genotype Nestin^TVA^ p21^–/–^ Ink4A/ARF^fl/fl^ Atm^fl/fl^ (nIp21A^fl/+^) (Figure 5A). The time to tumor formation was similar regardless of the presence of intact *Atm* (Figure 5B). Surprisingly, p21-null mice bearing tumors with *Atm* deletion had shorter survival following fractionated focal brain irradiation compared with their littermate controls with intact *Atm* in the tumors (*P* < 0.03, by log-rank test; Figure 5C). We confirmed p21 loss using IHC (Figure 5, D–E). We found that p21-null tumors with and without *Atm* loss had similar proliferation indices, as assessed by Ki67 staining (Figure 5F). TUNEL staining of irradiated tumors showed that *Atm* loss was associated with significantly increased TUNEL staining (*P* < 0.05, Figure 5, G and H), suggesting that tumors lacking both *Atm* and *Cdkn1a* were primed for apoptosis. These results show that functional *Cdkn1a* was not a key mediator of radiosensitization by *Atm* loss in the primary mouse model of DMG.

### A p53 transactivation domain mutant retains its tumor suppressor function in mouse DMG.

**Figure 6:**
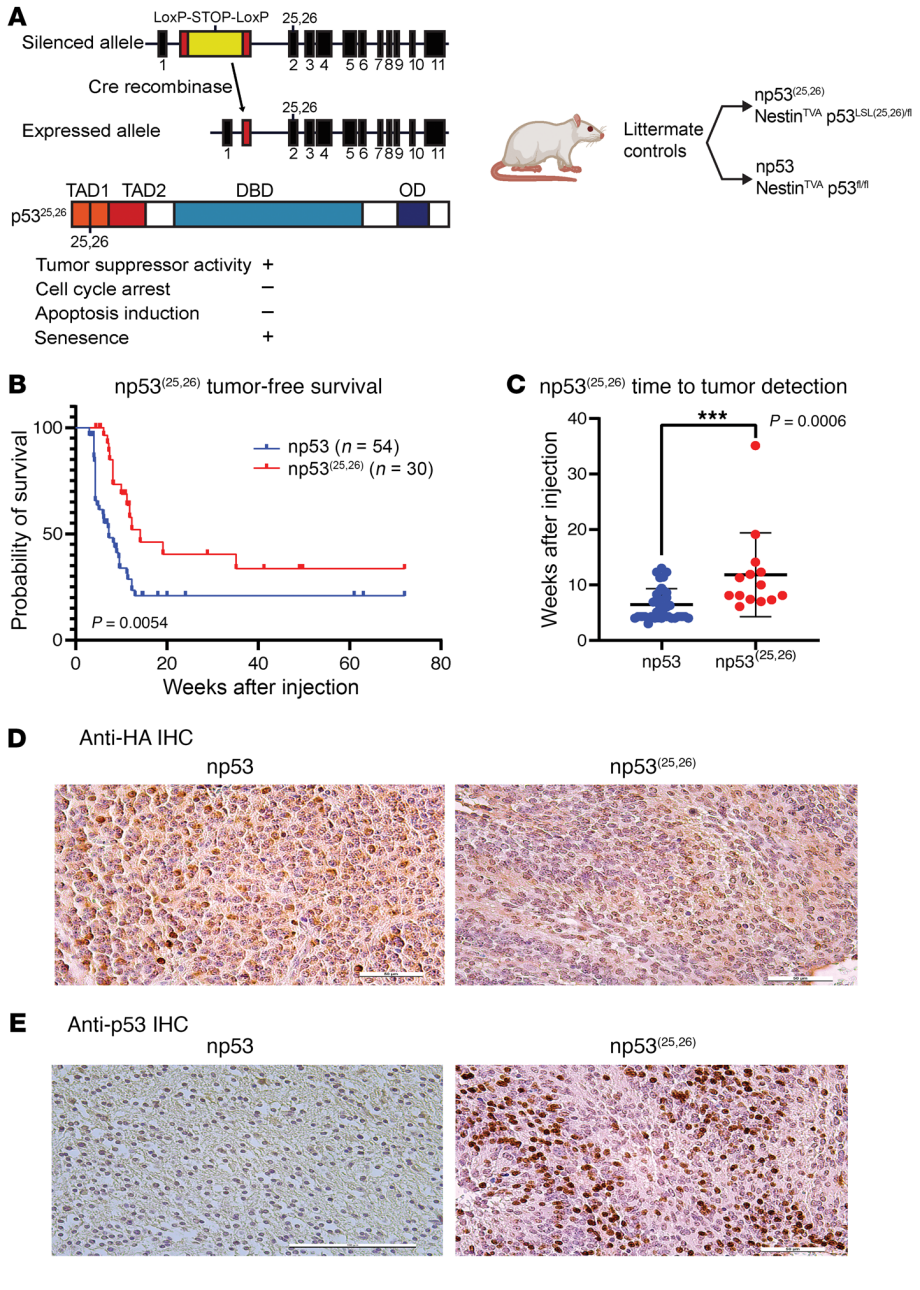
Tumor formation in mice expressing a p53 transactivation domain 1 mutant. (**A**) Schematic for conditional the p53 transactivation domain 1 mutant and mouse genotypes for expression of a p53 transactivation domain 1 mutant. (**B**) Tumor-free survival in the np53^(25,26)^ group compared with the np53 control group based on a log-rank test. (**C**) Time to tumor presentation in the p53^25,26/fl^ group compared with the p53^fl/fl^ control group (Wilcoxon test). (**D**) IHC for anti-HA staining in the p53 and p53^(25,26)^ groups. Scale bars: 50 μm. (**E**) IHC for p53 expression in the p53 (scale bar: 100 μm) and p53^(25,26)^ (scale bar: 50 μm) groups.

Since *Atm* loss could not radiosensitize *Cdkn1a*-null DMGs, we reasoned that regulation of p53 transcriptional targets other than *Cdkn1a* may cause radioresistance in WT p53, *Atm*-null DMGs. To investigate this possibility, we leveraged the conditional *loxP-Stop-loxP-p53^25,26^* allele (p53^LSL-25,26^) (34). In the presence of Cre recombinase, this allele expresses p53^25,26^, a p53 mutant that is severely compromised for the transactivation of most p53 target genes and cannot induce G_1_ arrest or apoptosis in response to acute DNA damage (34). Interestingly, p53^25,26^ retains tumor suppressor activity in lung tumors (34), however it is unknown whether it retains tumor suppressor activity in brain tumors. We first determined whether p53^25,26^ retains tumor suppressor activity in DMG. To test this hypothesis, we compared littermate mice with either p53^LSL-25,26/fl^ or p53^fl/fl^ mice. All mice harbored Nestin^TVA^ and were injected with Cre, luciferase, and PDGF-β retrovirus constructs as described above (Figure 6A). We noted a marked delay in tumor presentation in the p53 ^LSL-25,26/fl^ group compared with that in the p53^fl/fl^ controls (Figure 6, B and C). Immunohistochemical analysis revealed heterogeneous p53 expression in the p53^LSL-25,26/fl^ group and apparently absent p53 expression in the p53^fl/fl^ group (Figure 6, D and E). Thus, a p53 mutant with severely compromised transactivation activity retained its tumor suppressor activity in primary mouse brainstem gliomas. These results indicate that p53 transactivation function is dispensable for p53 tumor suppression in DMG.

### Atm loss does not radiosensitize mouse DMGs lacking a functional p53 transactivation domain.

**Figure 7:**
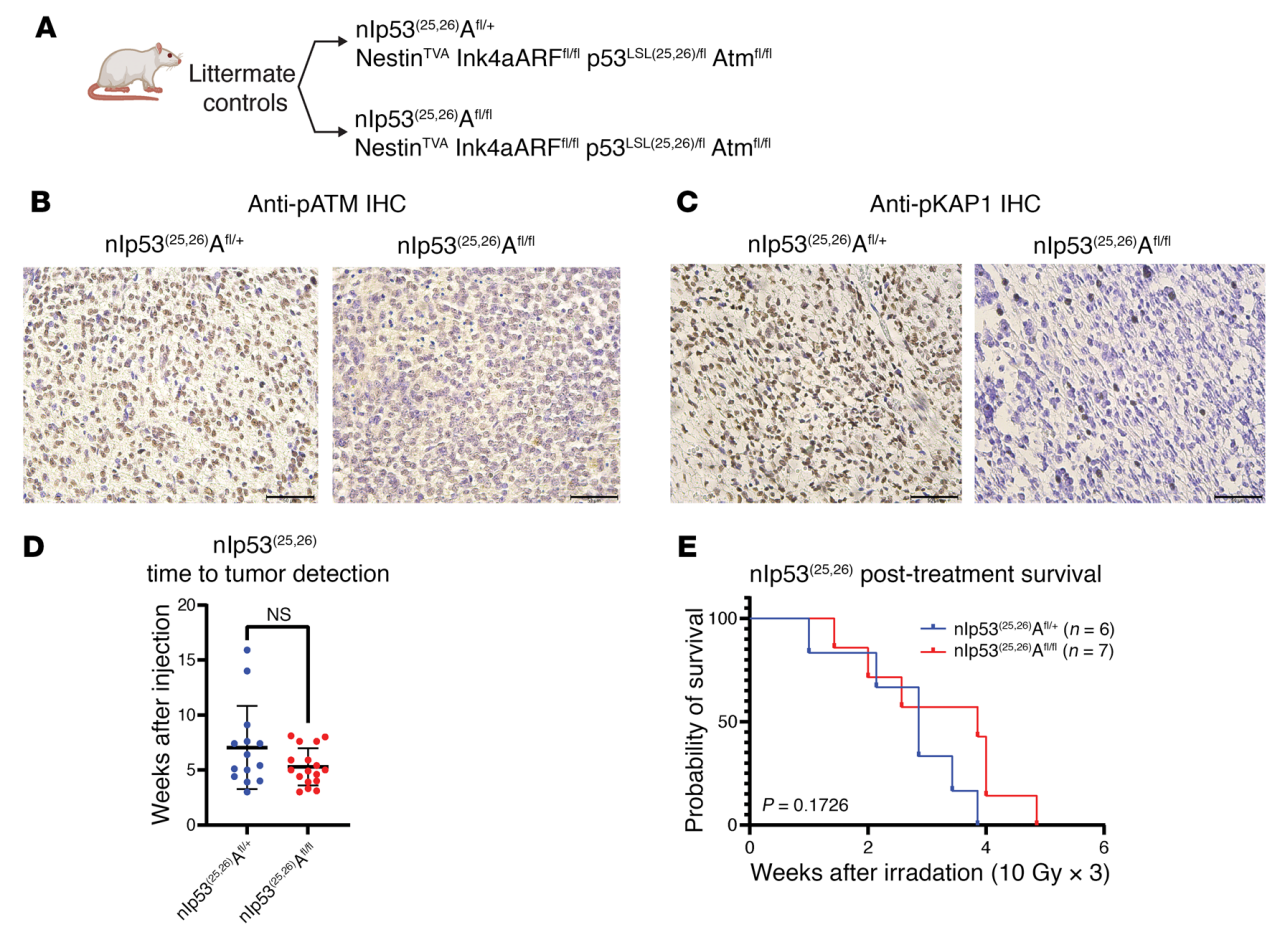
Effect of *Atm* loss on survival after fractionated focal brain irradiation in mouse DMGs expressing a p53 transactivation domain 1 mutant. (**A**) Schematic showing the p53^LSL(25,26)^ allele and genotypes for Nestin^TVA^ p53^LSL(25,26)/fl^ Ink4A/ARF^fl/fl^ mice with either Atm^fl/fl^ or Atm^fl/+^. (**B**) IHC images showing p-Atm in Atm^fl/+^ and Atm^fl/fl^ tumors. Scale bars: 50 μm. (**C**) IHC images showing p-KAP1 expression in Atm^fl/+^ and Atm^fl/fl^ tumors. Scale bars: 50 μm. (**D**) Time to tumor formation in Nestin^TVA^ p53^LSL(25,26)/fl^ Ink4A/ARF^fl/fl^ mice with either Atm^fl/fl^ or Atm^fl/+^ (dot plot). NS, by Wilcoxon test. (**E**) Overall survival following fractionated brain irradiation in mouse DMGs expressing a p53 transactivation domain 1 mutation with or without *Atm* loss. The *P* value is based on a log-rank test.

We next sought to determine whether *Atm* loss could radiosensitize DMGs lacking p53 transcriptional activity but retaining other nontranscriptional functions of p53. We previously showed that *Atm* loss did not radiosensitize brainstem gliomas driven by Ink4A and ARF loss, however *Atm* loss modestly radiosensitized brainstem gliomas with both Ink4A and ARF loss and p53 loss (5). We reasoned that if loss of p53 transactivation domain function is the determinant of radiosensitization by *Atm* loss, then brainstem gliomas with both Ink4A and ARF loss and expression of a transactivation-deficient p53^25,26^ allele would be radiosensitized by *Atm* loss. To test if mouse DMGs with p53^25,26^ and Ink4A and ARF loss were radiosensitized by *Atm* loss, we bred mice of the Nestin^TVA^ p53^LSL-25,26/fl^ Ink4A/ARF^fl/fl^ Atm^fl/fl^ genotype. To test the effects of *Atm* loss, we compared these mice with their littermate controls with the same genotype except an intact *Atm* allele (Nestin^TVA^ p53^LSL-25,26/fl^ Ink4A/ARF^fl/fl^ Atm^fl/+^) (Figure 7A). We noted similar time to tumor formation in both models (Figure 7D). *Atm* loss was associated with differential staining of p-ATM and p-KAP1 after focal brain irradiation (Figure 7, B and C), confirming the loss of ATM functional activity. After subjecting the mice to fractionated focal brain irradiation, no difference in overall survival was appreciated (Figure 7E). These results indicate that the transactivation-independent functions of p53 may be the primary determinants of whether mouse DMGs can be radiosensitized by *Atm* loss.

## Discussion

Here, we describe the generation of primary mouse DMGs based on recent advances in murine genetic engineering including the conditional H3.3K27M allele and the RCAS/tv-a retrovirus platform (10, 13, 14). We used this model to show that the genetic loss of *Atm*, an important target for drugs that have entered clinical trials for patients with brain tumors (18), radiosensitizes primary DMG models. Our results in p53-null/H3.3K27M mouse DMGs were similar to those reported previously for p53-null mouse brainstem gliomas (5), in which the sole difference was the presence of H3.3K27M expression from the endogenous *H3f3a* locus in the neoplastic tumor cells in our current model. In addition, we generated several unique genetically engineered mouse models with differential responses based on genotype (highlighted in Table 1), which suggests that H3.3K27M is not a primary determinant of the ability to target ATM to enhance the efficacy of RT in primary mouse DMG models (Supplemental Figure 13).

Our results from genetic experiments in primary mouse models indicate that p53 is a key determinant of the ability of DMGs to be radiosensitized by *Atm* loss. Almost all p53-altered primary mouse models were radioresistant and radiosensitized by *Atm* loss, including (a) a model driven by p53 loss with WT *H3f3a* (5); (b) a model driven by both p53 loss and loss of Ink4A/ARF(5); and (c) the H3.3K27M/TP53 mutant model reported here (Figure 1E). In contrast, *Atm* loss is unable to radiosensitize primary p53-WT brainstem glioma mouse models, including models driven by Ink4A and ARF loss (5) and models driven by *Pten* loss (11). Notably, a recent study comprehensively found that pharmacological ATM inhibition radiosensitizes both p53-mutant and WT p53 patient-derived models of DMG and pediatric high-grade glioma (6). Also, H3.3K27M may enhance ATM signaling, increasing radiosensitivity with and without ATM inhibition (35). Together, these data suggest that the mutational status of p53, H3.3K27M, and other alterations should be tested in correlative analyses in future clinical trials of ATM inhibitors in patients with DMG.

Our ISS data provide what we believe to be the first high-resolution transcriptional analysis at high gene plexy (~300 gene targets) in a mouse tumor model, which is critical to defining the model’s tumor vasculature and neoplastic compartments that play distinct roles in the therapeutic response (12). Future work will leverage these data to interrogate tumor immune and vascular microenvironment alterations induced by irradiation and *Atm* loss, which may guide the rational design of combinations of RT, ATM inhibitors, and therapies targeting the immune system or vasculature.

This work has several limitations. H3.3K27M did not decrease tumor latency in our system, as has been observed in other experimental systems (36). This may be due to the highly restricted manner of H3.3K27M induction in our system (i.e., from the endogeneous *H3f3a* locus and only in spatially and lineage-restricted cells) and/or the use of a relatively strong PDGF-β co-driver alteration that could mask more subtle H3.3K27M driver phenotypes in our system. Also, the presence of an HA tag on both HA–PDGF-β and H3.3K27M-FLAG-HA constructs precludes specific identification of PDGF-β in the nPH system. Finally, we observed a trend toward improved overall survival with ATM inhibition in an *Atm*-intact genetic mouse model. We hypothesize that the heterogeneity in tumor latency and the timing of treatment delivery may have conferred a significant survival benefit that was difficult to detect compared with the genetic loss of *Atm* and compared with the pharmacological xenograft experiment. Future studies could interrogate ATM inhibition effects in *Atm*-null models to discern on-target versus off-target effects of the ATM inhibitor.

The current work implicates transactivation-independent mechanisms by which p53 mediates radioresistance in *Atm*-null tumors. Our ISS data show that irradiation elicited overexpression of *Cdkn1a*, a key downstream target of p53 that mediates cell senescence and G_1_-to-S checkpoint arrest, in p53-null tumors, indicating p53-independent mechanisms of *Cdkn1a* expression (37). This finding led us to dissect the contribution of p53 transactivation functions that regulate p21 expression to radiosensitization in *Atm*-null DMGs. While *Atm* loss radiosensitizes tumors lacking p53, we found that *Atm* loss could not radiosensitize tumors containing a p53^25,26^ allele deficient in p53 transactivation function. Similarly, tumors lacking *Cdkn1a* (p21) could not be radiosensitized by *Atm* loss. Our findings highlight the importance of carefully considering p21 status in clinical trials involving Atm inhibition, given the complex role of p21 in tumor growth and the microenvironment (38). Strikingly, we found that *Atm* loss made tumors more radioresistant in mice that lacked *Cdkn1a* and that this loss was associated with increased apoptosis. These findings implicate the transactivation-independent function of p53 as a key determinant of radiosensitivity in *Atm*-null tumors. Future work will dissect the transactivation-independent functions of p53, such as the promotion of apoptosis through mitochondrial membrane permeabilization, direct repression of transcription, and/or direct interaction with complexes that detect DNA lesions (39, 40). Our data provide genetic and mechanistic insights that build upon studies of pharmacological ATM inhibition in patient-derived xenograft models (6). In addition, our work shows that ATM inhibition improves the response to irradiation, leading to extended survival. Further studies are needed to determine the transactivation-independent mechanisms of p53 and ATM-directed therapies and their effect on overcoming resistance to RT in patients with H3.3K27M-mutant DMG.

## Methods

### Sex as a biological variant.

Male and female mice were used in all murine experiments to ensure representation of both sexes. We did not identify any sex-specific differences in the data, and all findings were consistent across male and female mice. As such, sex was not considered to be a biological variable in the interpretation of the results. The outcomes of this study are therefore expected to be broadly relevant to both sexes.

Detailed workflows for the generation, brain irradiation, and molecular analysis of primary mouse DMG models using RCAS/tv-a and Cre/loxP technologies are described in our recent publication (41). Male and female mice were utilized for all murine models. All new reagents, materials, and software are listed in and software are listed in Supplemental Table 8. A list of the abbreviations used is provided in Supplemental Table 7.

### Mouse strains.

Detailed workflows for generation, brain irradiation, and molecular analysis of primary mouse DMG models using RCAS/tv-a and Cre/loxP technologies are found in our recent *STAR Protocols* publication (41). Complex mouse strains were generated by breeding mice with the following alleles: *Nes^TVA^*, *Atm^fl^*, and *p53^fl^* (41), *Ink4A/ARF^fl^* (41), *p53^LSL(25,26)^* (34), and *p21^–/–^* (42). The *H3f3a^LSL-K27M-Tag^* allele was a gift from Suzanne Baker, St. Jude Children’s Research Hospital, Memphis Tennessee (10).

### DF1 cell culture and retrovirus generation.

DF1 cells were cultured in DMEM containing 10% FBS, and RCAS/tv-a retroviruses were generated using RCAS-Cre, RCAS-luc, and RCAS-PDGF-β plasmids as previously described (41).

### Mouse brainstem injection.

The harvested DF1 cells were injected into the brainstems of mice anesthetized on ice on P3–P5 as previously described (41). Patient-derived xenografts using the SF8628 model were generated by brainstem injection as described before (29–31).

### Mouse in vivo imaging.

Bioluminescence imaging of gliomas in mice was performed by intraperitoneal injection of d-luciferin and IVIS Illumina III as previously described (41).

### Image-guided focal brain irradiation.

Irradiation of gliomas in mice was delivered on a SARRP using image-guided, opposed-lateral beams as described previously (41). Daily fractions of 10 Gy were delivered for primary DMG models on 3 consecutive days. For patient-derived xenograft DMG models, a dose of 2 Gy was delivered 3 days a week, for a total dose of 12 Gy.

### ATM inhibitor studies.

For the patient-derived xenograft model, SF8628 (H3.3K27M DIPG) was obtained from the UCSF Medical Center. Establishment of SF8628 cell culture from surgical specimens and tumor cell modification for expression of firefly luciferase for in vivo bioluminescence imaging have been described in previous publications (30–32). SF8628 cells were propagated as monolayers in complete medium consisting of DMEM supplemented with 10% FBS and nonessential amino acids. Short tandem repeats (STRs) were obtained to confirm the identity of the cell lines. All cells were cultured in an incubator at 37°C in a humidified atmosphere containing 95% O_2_ and 5% CO_2_ and were free of mycoplasma at the time of testing. Six-week-old female athymic mice (rnu/rnu genotype, BALB/c background) were purchased from Envigo and housed under aseptic conditions. Pontine injection of tumor cells was performed as previously described (29–31). Each mouse was injected with 1 μL SF8628 cell suspension (100,000 cells/μL) into the pontine tegmentum at a depth of 5 mm from the inner base of the skull. For the efficacy study of AZD1390 and radiation, animals were randomized into 4 treatment groups: (a) vehicle control (0.5% hydroxymethylcellulose, 0.1% Tween 80, *n* = 6); (b) ADZ1390 monotherapy (oral gavage of 20 mg/kg AZD1390 for 5 times per week for 2 consecutive weeks, *n* = 6); (c) radiation monotherapy (2.0 Gy, 3 times per week for 2 consecutive weeks for a total dose of 12 Gy, *n* = 6); and (d) AZD1390 and radiation combination therapy (*n* = 6). Biweekly bioluminescence imaging was used to monitor tumor growth and response to therapy as previously described (29). Mice were monitored daily and euthanized at the endpoint, which included irreversible neurological deficit or a body condition score of less than 2. For the genetically engineered mouse model, gliomas were generated using the RCAS/Tv-a system as previously described (43, 44). DF1 cells were transfected with RCAS plasmids (RCAS–PDGF-β, RCAS-Luc, and RCAS-Cre, mixed at a 1:1:1 ratio prior to intracranial injections). nP mice were intracranially injected with 1.0 μL RCAS virus producing DF1 cells at P3 to 5 (P3–P5) and monitored 3 times per week after weaning for signs of brain tumor symptoms (enlarged head, ataxia, weight loss up to 20%). Bioluminescence imaging was then used to monitor tumor formation from weeks 4–12 as described previously (38). Following tumor detection via imaging or the onset of neurological symptoms, mice were randomized 1:1 into 2 treatment groups: RT alone or AZD1390 plus RT. AZD1390 was obtained from AstraZeneca and resuspended in 0.5% w/v is hydroxypropyl methylcellulose and 0.1% w/v Tween 80 solution. Mice in the AZD1390 plus RT group were dosed at 20 mg/kg via oral gavage 1 hour prior to RT, as suggested by the superior efficacy dose (18). Both treatment groups received 3 consecutive daily fractions of 10 Gy focal brain irradiation delivered by the SARRP. Treated mice were monitored for survival until they reached the humane endpoint. (14, 18)

### IHC.

IHC analysis was performed using methods previously described for Ki67, p-Atm, p-KAP1, total KAP1, γH2AX, and p53 (18, 41). Additional IHC and TUNEL staining was performed by HistoWiz (histowiz.com) using a standard operating procedure and a fully automated workflow for p21 (Cdkn1a), FLAG, and TUNEL on a Bond Rx autostainer (Leica Biosystems) with enzyme treatment (1:1,000) using standard protocols. H&E staining was performed using standard protocols.

### Xenium in situ and bioinformatics analysis.

Tumor-bearing brains subjected to Xenium ISS were detected by in vivo imaging 37–48 days after birth and collected 7 days after tumor detection, either after 3 daily treatments of 10 Gy initiated within 2 days of tumor detection or after mock treatment. Initial data generated by the Xenium instrument (45) are processed on board with a built-in analysis tool called the Xenium Analyzer (45). The Xenium Analyzer is fully automated and includes an imager (imageable area of approximately 12 × 24 mm per slide), sample handling, liquid handling, wide-field epifluorescence imaging, capacity for 2 slides per run, and an on-instrument analysis pipeline. The analysis pipeline included image preprocessing, puncta detection, transcript decoding, and quality score assignment. The pipeline also performed cell segmentation using DAPI images to detect nuclei using a neural network. Each nucleus was then expanded outward until either a maximum distance of 15 μm was reached or the boundary of another cell was reached. A variety of output files were produced using an on-instrument pipeline. The essential files for downstream analysis included the feature-cell matrix (HDF5 and MEX formats identical to those output by single-cell RNA tools from 10x Genomics [Cellranger/Spaceranger]), the transcripts (listing each mRNA, its 3D coordinates, and a quality score), and the cell boundaries CSV file.

Xenium output was first imported into R (4.3.1) using the LoadXenium function from Seurat (version 4.9.9.9050) (46). The 4 Xenium samples were processed using Seurat (46). Data were loaded and filtered using an nFeature_Xenium>5 and nCount_Xenium of greater than 10 as a criterion. Cells without a predicted annotation were then subset out, and the 4 samples were normalized using SCTransform. Principal component analysis was also run for each sample. The 4 samples were then integrated using the IntegrateLayers function, with HarmonyIntegration as the method (47).

Specific regions of the tissue were annotated manually using the polygon tool in Xenium Explorer software (development version, 10x Genomics), and the polygon coordinates were exported as.csv files. Cells with zero counts were then filtered, and the points within the polygon coordinates were identified using the point.in.polygon function in the sp (2.0.0) R package. Additional plots were generated using Seurat, and deconvolution was performed with spacexr (version 2.2.1) (48) using a custom annotated single-cell reference from a previous experiment. Differential gene expression analysis across regions of interest (ROIs) was assessed with the Wilcoxon test on SCTransform normalized count data using the FindMarkers Seurat function. Co-occurrence and neighborhood enrichment plots were generated using the Python package Squidpy (version 1.2.3) (49), and trajectory and cell-cell-interaction analyses were performed using the Python package STLearn (version 0.4.12) (50).

All the cells on the entire slide were used to determine the different types of cell clusters. All unlabeled cells were removed for Squidpy and STLearn analyses. Co-occurrence and neighborhood enrichment analyses were conducted on all cells within the entire slide. The tumor core and periphery were utilized to identify cell-cell and cell-ligand interactions. Differentially expressed genes were analyzed in the tumor core.

### Whole-genome bisulfite sequencing and bioinformatics analysis.

Bisulfite methylation sequencing and data analysis were performed by Novogene. Briefly, K27M-mutant (nPH) and matched WT K27M (nP) tumor-bearing mice (*n* = 4 biological replicates per group) were generated as described above, and tumor-bearing brains were embedded in FFPE. Tumor regions were identified in the brains on matched H&E-stained slides, and tumor microdissection was performed. Genomic DNA was isolated, spiked with lambda bacteriophage DNA (to serve as an internal negative control), and fragmented to 200–400 base pairs, and bisulfite treatment was performed to convert unmethylated cytosines into uracil via deamination. Notably, this process does not alter methylated cytosines, allowing identification of these sites downstream. After methylation sequencing adapter ligation, dsDNA synthesis, and library size selection, PCR amplification was performed followed by Illumina sequencing. FastQC was used for quality control on the raw reads. Bismark software (version 0.24.0) was used to perform alignments of bisulfite-treated reads to a reference genome (-X 700--dovetail). Bismark software is a tool utilized for bisulfite sequencing data analysis and the source code is freely available. The reference genome was firstly transformed into a bisulfite-converted version (C-to-T and G-to-A converted) and then indexed using bowtie2, a software tool utilized for aligning sequencing reads to the reference genome. Sequence reads were also transformed into fully bisulfite-converted versions (C-to-T and G-to-A converted) before they were aligned to similarly converted versions of the genome in a directional manner. Sequence reads that produced a unique best alignment from the 2 alignment processes (original top and bottom strand) were then compared with the normal genomic sequence, and the methylation state of all cytosine positions in the read was inferred. The same reads that aligned to the same regions of the genome were regarded as duplicated ones. The sequencing depth and coverage were summarized using deduplicated reads. The results of the methylation extractor (bismark_methylation_extractor,-- no_overlap) were transformed into the bigWig format for visualization using the Integrative Genomics Viewer (IGV) browser. The sodium bisulfite nonconversion rate was calculated as the percentage of cytosine sequenced at cytosine reference positions in the lambda genome. Genes were extracted from genome assembly GRCm39 using Ensembl gene set version 111. Promoter regions were defined from 1,500 bp upstream of the transcription start site (TSS) to 500 bp downstream. For each region, methylated CpG reads and unmethylated reads were counted and summed, and the average methylation level was calculated. Similarly, putative enhancers were extracted from Ensembl regulation version 111 of GRCm39, and an average methylation level was calculated for each enhancer. The methylation difference was determined for each feature (promoter and enhancer) between samples from the K27M group and samples from the nPA group. Significance was estimated by applying ANOVA over a linear mode fit (51). For DNA methylation extraction, the methikit was used. Motif analysis was carried out using HOMER (http://homer.ucsd.edu/homer). For pathway enrichment analysis, STRING (https://string-db.org/) was used. For “other tissues” we analyzed data from the NCBI Gene Expression Omnibus (GEO) database (GSE42836). Methylation extraction was done using the methkit.

### Statistics.

Statistical significance in volcano plots across ROIs was assessed with the Wilcoxon test on SCTransform-normalized count data using the FindMarkers Seurat function. Data plotting and quantification analyses were performed using GraphPad Prism 9 (GraphPad Software). An unpaired 2-tailed *t* test, Mann Whitney test, and/or one way ANOVA was utilized to determine significance in IHC quantifications was utilized to determine significance in IHC quantifications. The log-rank test was used to determine the survival rate. The Wilcoxon test was used to determine differences in the time to tumor detection. Individual data points are plotted, and all statistically significant values (*P* < 0.05) are identified with an asterisk. Data points are presented as the mean with SD.

### Study approval.

All animal studies were conducted according to institutional animal protocols. All animal experiments were approved by the IACUCs of Duke University and Northwestern University. For the patient-derived xenograft model, the human cell line SF8628 (H3.3K27M DIPG) was obtained from the UCSF Medical Center, in accordance with an institutionally approved protocol.

### Data availability.

All data were deposited in the NCBI GEO database (GSE246584; for whole-genome bisulfite sequencing, GSE284759). Additional information required to reanalyze the data reported in this work is available from the corresponding author upon request. All raw values for figures are available in the Supporting Data Values file.

## Author contributions

AM prepared the manuscript, designed experiments, and analyzed data. VV designed and led the execution of experiments for the revised manuscript including mouse tissue analyses, whole-genome bisulfite sequencing, and pharmacologic in vivo experiments. AM and VV share the first author position. The order of the first authors’ names was determined by alphabetical order of the last names (the order was decided to be A–Z on the basis of a coin-flip by the corresponding author ZJR). AM, ZJR, SGG, DGK, DMA and OJB designed the study and experiments. SRW, HQL, BEF, LBW, MEGG, DGK, LL, KD, VV, SMM, MR and ZJR performed mouse experiments and tabulated data. NTW performed mouse irradiation procedures. K Abe, EU, and RH performed patient-derived xenograft and ATM inhibitor pharmacologic experiments. LA contributed the p53 transactivation mutant mouse strain and experimental design regarding the strain. EH, K Abramson, LBW, and ZJR assisted with in situ sequencing experiments. MA, VJ, JAR, SG and ZJR performed bioinformatics analyses. TFM performed whole-genome bisulfite sequencing analyses. CES performed mouse experiments and tabulated data. AM, ZJR, and SGG prepared the manuscript.

## Supplementary Material

Supplemental data

Unedited blot and gel images

Supplemental table 1

Supporting data values

## Figures and Tables

**Table 1:**
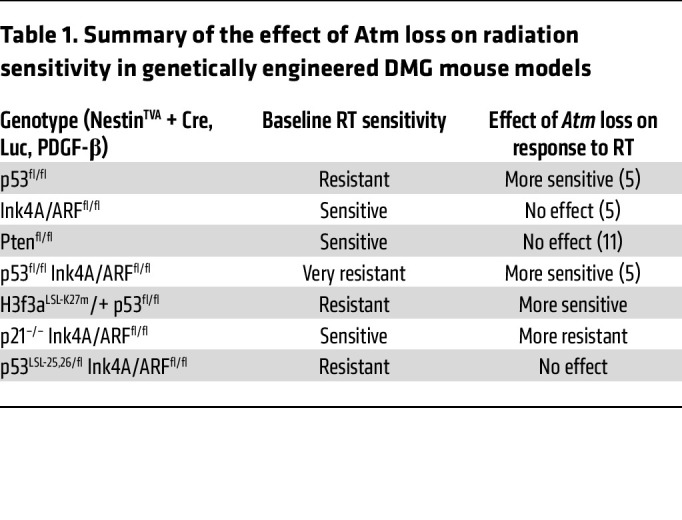
Summary of the effect of Atm loss on radiation sensitivity in genetically engineered DMG mouse models

## Version 1 04/17/2025

In-Press Preview

## Version 2 06/16/2025

Electronic publication
