# Supplementary material for: Disruption of ataxia telangiectasia–mutated kinase enhances radiation therapy efficacy in spatially directed diffuse midline glioma models
Source: J Clin Invest. 2025 Apr 17;135(12):e179395. doi: 10.1172/JCI179395 (PMC12165813; doi:10.1172/JCI179395)
Supplement: Supplemental data [file jci-135-179395-s055.pdf]

SUPPLEMENTAL FIGURES AND SUPPLEMENTAL TABLES

Supplemental Figure 1 - Trimethylation H3K27me3 staining quantification in primary mouse DMG H3.3K27M ($Nestin^{TVA}$; $p53^{FL/FL}$; $H3f3a^{loxP\text{-}Stop\text{-}loxP\text{-}K27M\text{-}Tag/+,}$ nPH) with or without conditional H3.3K27M allele

Supplemental Figure 2 – Genome wide methylation between with and without H3K27M alteration in murine primary glioma model

Supplemental Figure 3 – UMAP Single Cell phenotyping of a representative tumor-bearing brain.

Supplemental Figure 4 – Differentially expressed marker genes for each cluster identified within all tumors (n=4).

Supplemental Figure 5 – Collapsed individual cell clustering into 10 archetypal cell types.

Supplemental Figure 6 – Violin plot with expression of Atm within Atm intact and Atm null tumors.

Supplemental Figure 7 – snRNA sequencing data from murine models to assess Semaphorin and p21 pathways

Supplemental Figure 8 – Neighborhood analysis of primary mouse DMGs comparing ATM tumoral loss and/or focal irradiation.

Supplemental Figure 9 – Spatial Cell Interactions within tumor environment of primary mouse DMGs with tumoral Atm loss with/without focal irradiation

Supplemental Figure 10 - ATM variants from RNA sequencing data using the GATK for patient derived cell line SF8628

Supplemental Figure 11 – Survival curve of ATM intact genetically engineered murine glioma model with ATM inhibitor +/- irradiation

Supplemental Figure 12 – Raw images for western blot for DNA damage signaling in patient derived cell line, SF8628

Supplemental Figure 13 - Concurrent ATM and p53 loss in GEMMs drive sensitivity to radiation therapy

Supplemental Tables

Supplemental Table 1 – Panel of 298 mouse brain and DMG transcripts targeted by in situ sequencing. (See separate Excel File).

Supplemental Table 2 – Collapsed individual cell clustering into 10 archetypal cell types.

Supplemental Table 3 –Top differentially expressed genes of *Atm* intact (FL/+) with and without irradiation.

Supplemental Table 4 –Top differentially expressed genes of *Atm* null (FL/FL) with and without irradiation.

Supplemental Table 5 – Top Cell Ligand receptors with a p-value < 0.05 for all tumors.

Supplemental Table 6 - ATM variant in patient derived cell line SF8628.

Supplemental Table 7 – List of abbreviations.

Supplemental Table 8 – Raw data values for all statistical plots (see separate Excel file).

Supplemental Figure 1

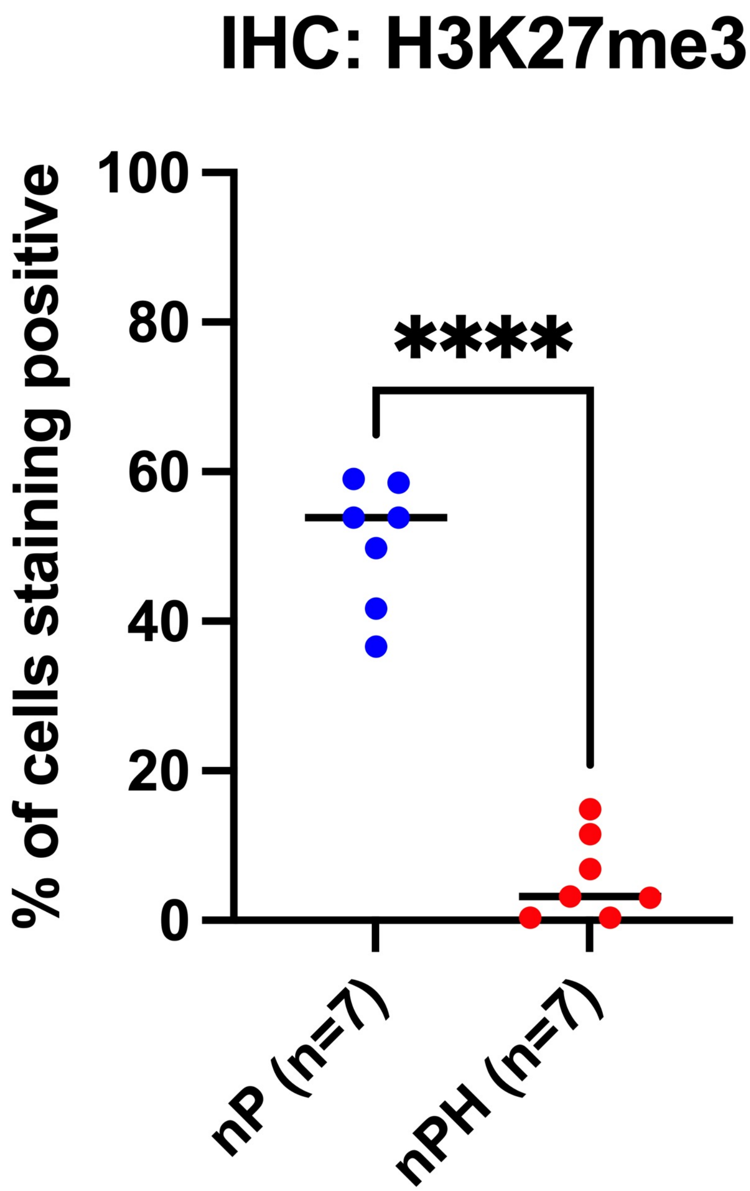

Supplemental Figure 1 - Trimethylation H3K27me3 staining quantification in primary mouse DMG H3.3K27M (Nestin^{TVA}; p53$^{FL/FL}$; H3f3a$^{loxP\text{-}Stop\text{-}loxP\text{-}K27M\text{-}Tag/+,}$ nPH) with or without conditional H3.3K27M allele

Quantification of H3K27me3 staining in nPHA $^{FL/+}$ mice (P-value < 0.0001) based on unpaired t-test in H3.3K27M (Nestin^{TVA}; p53$^{FL/FL}$; H3f3a$^{loxP\text{-}Stop\text{-}loxP\text{-}K27M\text{-}Tag/+,}$ nPH) model with or without conditional H3.3K27M allele (n =7 per group)

Supplemental Figure 2

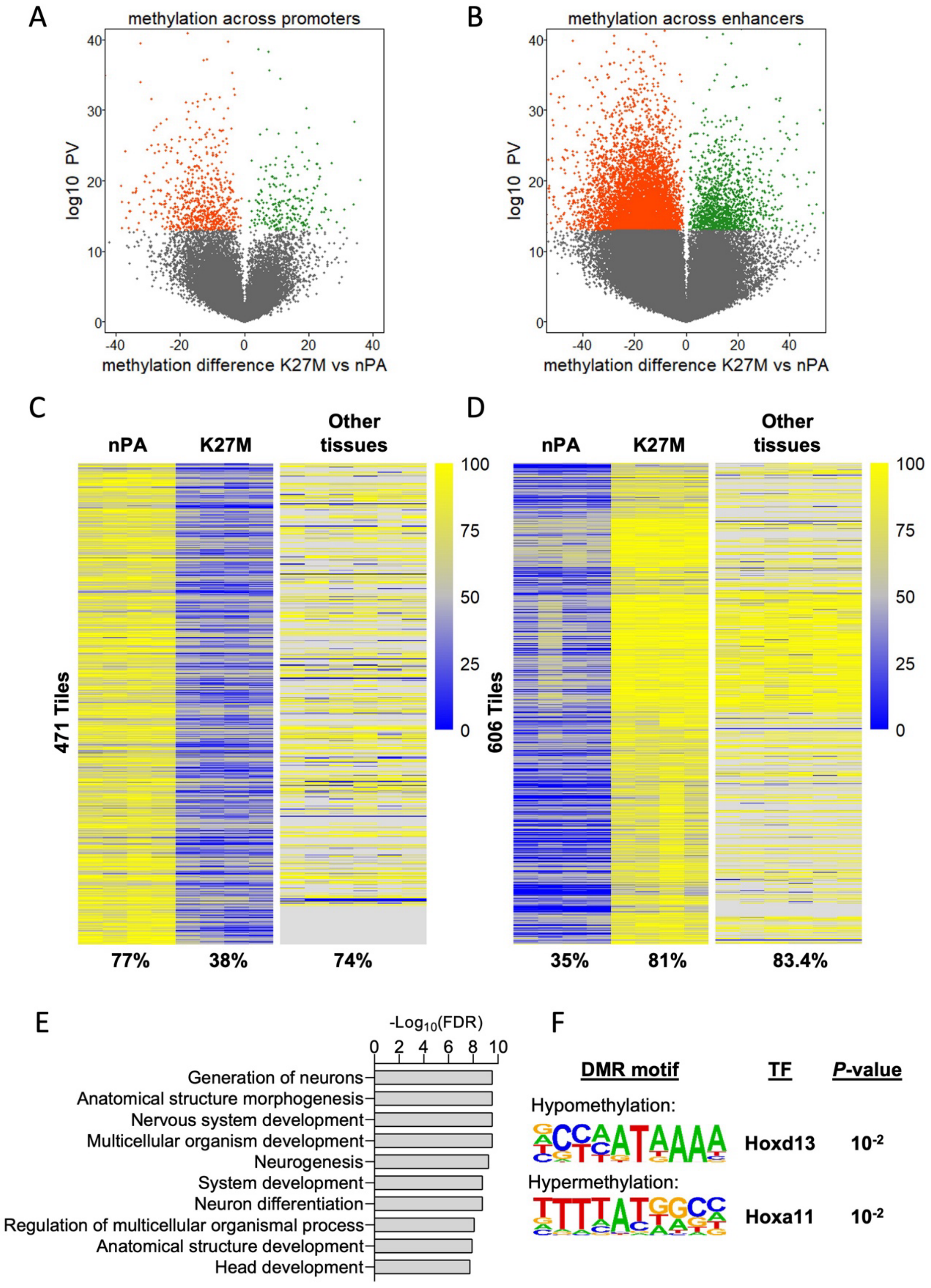

Supplemental Figure 2 - Genome wide methylation with and without H3K27M alteration in murine primary glioma model

(A) Volcano plot showing differentially methylated features in promoters results in 688 vs 232 promoters (p-value is 1.5E-7) tested against random distribution. Each dot in the volcano plots represents a feature. X-axis is methylation difference meth(K27M) – meth(nPA). Y-Axis is significance (log of p-value). Colored dots are top significant differentially methylated features (p-value < 0.05), hypo features are colored red and hyper green.

(B) Volcano plot showing differentially methylated features in enhancers results in 6649 vs 1309 enhancers (p-value 3.0E-90). Each dot in the volcano plots represents a feature. X-axis is methylation difference meth(K27M) – meth(nPA). Y-Axis is significance (log of p-value). Colored dots are top significant differentially methylated features (p-value < 0.05), hypo features are colored red and hyper green.

(C) Heatmap of at least 30% methylation difference for hypomethylated tiles (100bp) in K27M compared to nPA and other normal tissues (colon, kidney, skin, spleen, cerebellum and cortex).

(D) Heatmap of at least 30 % methylation difference for de-novo methylated tiles in K27M compared to nPA and other normal tissues (colon, kidney, skin, spleen, cerebellum and cortex).

(E) Pathway analysis for hypomethylated tiles based on nearest gene analysis.

(F) Motif analysis for hypo and hyper-methylated tiles.

Supplemental Figure 3

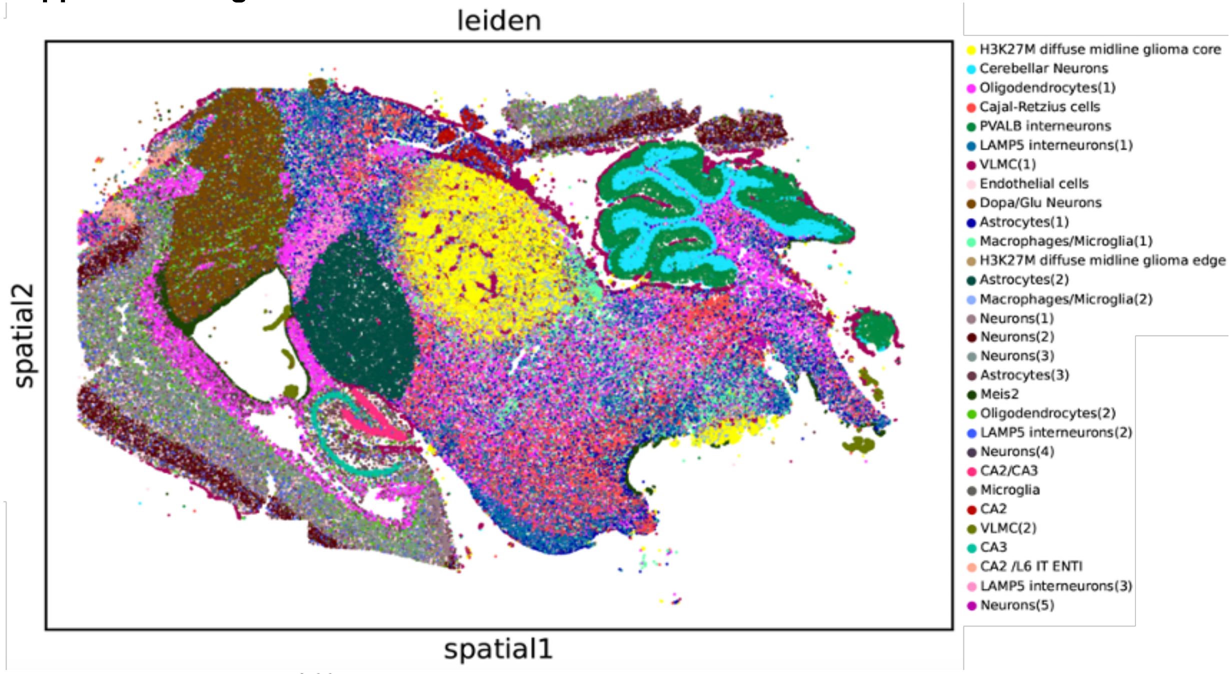

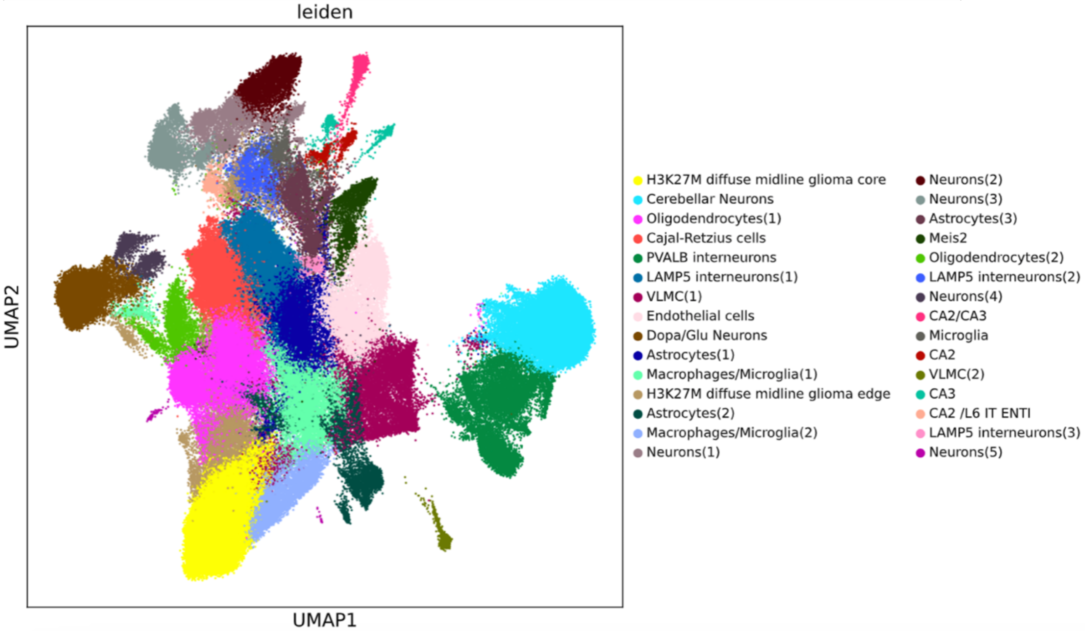

Supplemental Figure 3 - UMAP Single Cell phenotyping of a representative tumor-bearing brain.

Top - A sagittal section of an unirradiated mouse brain bearing a primary diffuse midline glioma containing H3.3K27M mutation and p53 and Atm loss (Nestin^{TVA}; p53$^{FL/FL}$; H3f3a$^{LSL\text{-}K27M\text{-}Tag}$; Atm$^{FL/FL}$; nPHA$^{FL/FL}$).

Bottom - UMAP Cell Cluster for the same sample. Tumor was generated by injection of chicken fibroblast cells producing Cre, luc, and PDGFB RCAS retroviral vectors. Cell types are inferred based on differentially expressed cell markers. At bottom, UMAP clustering of cells from the same sample.

Supplemental Figure 4

$Atm^{FL/+}$ ($nPHA^{FL/+}$)

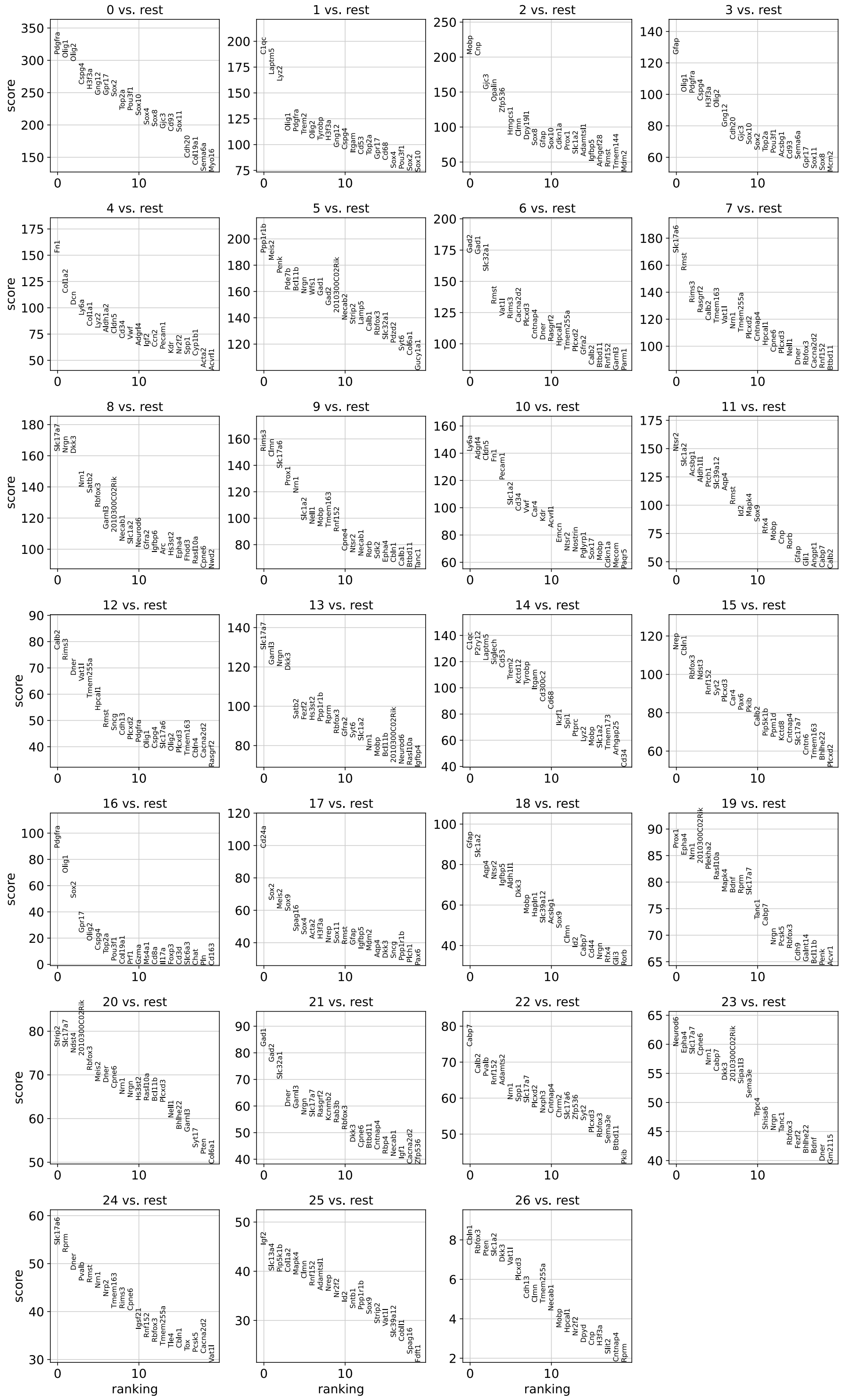

$Atm^{FL/+}$ ($nPHA^{FL/+}$) with irradiation (10Gy x 3)

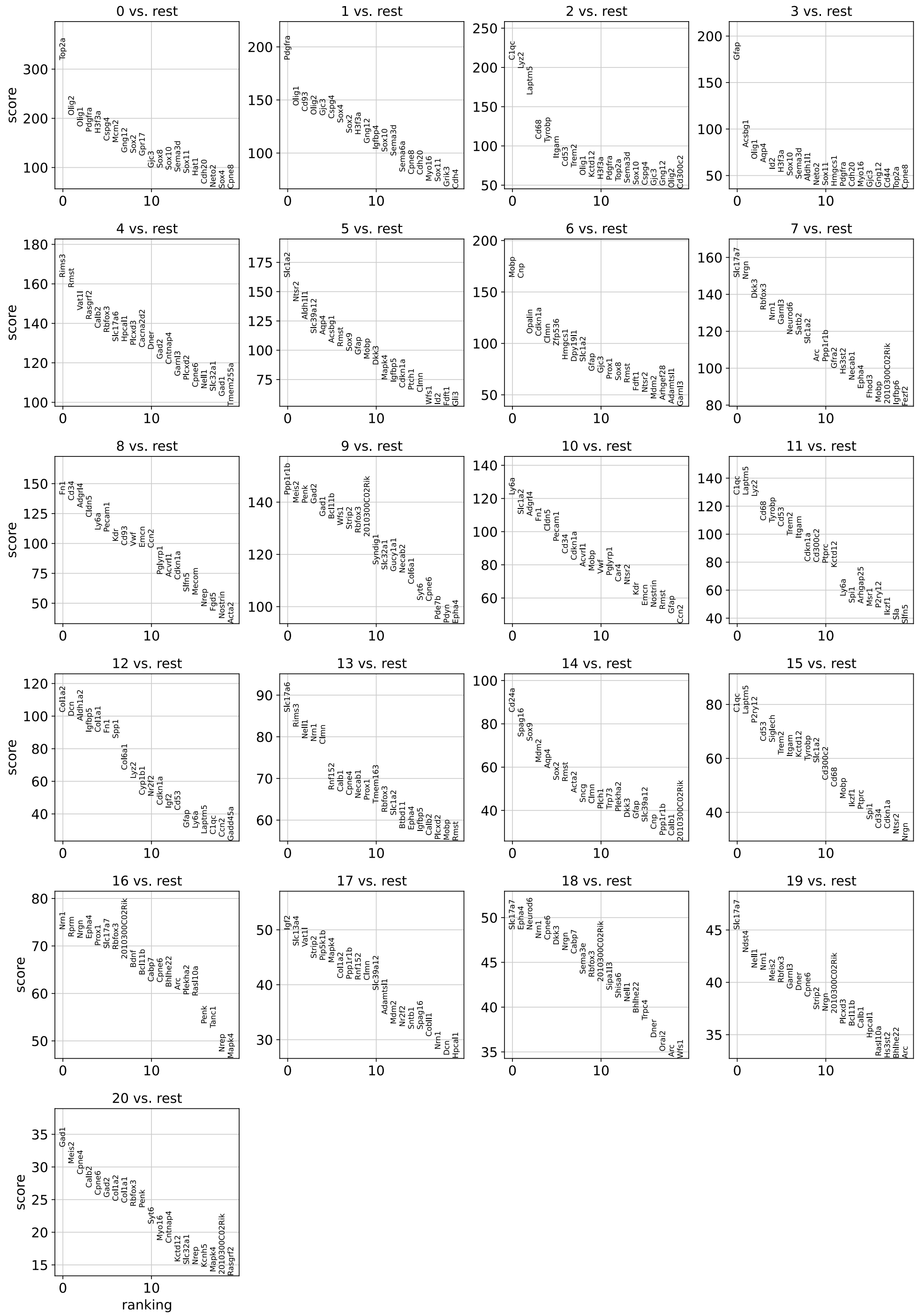

Atm$^{FL/FL}$ (nPHA$^{FL/FL}$)

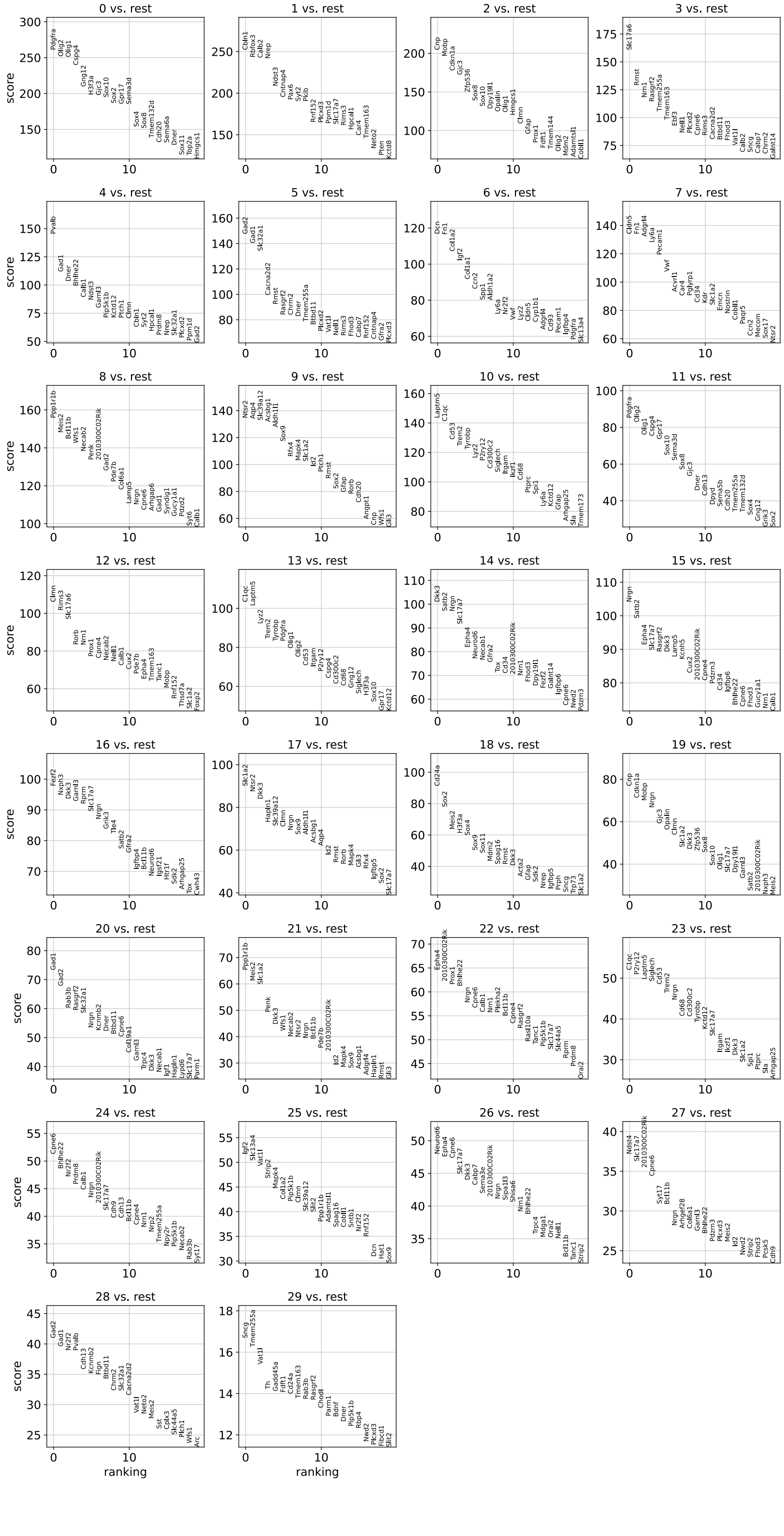

Atm$^{FL/FL}$ (nPHA$^{FL/FL}$) with irradiation (10Gy x 3)

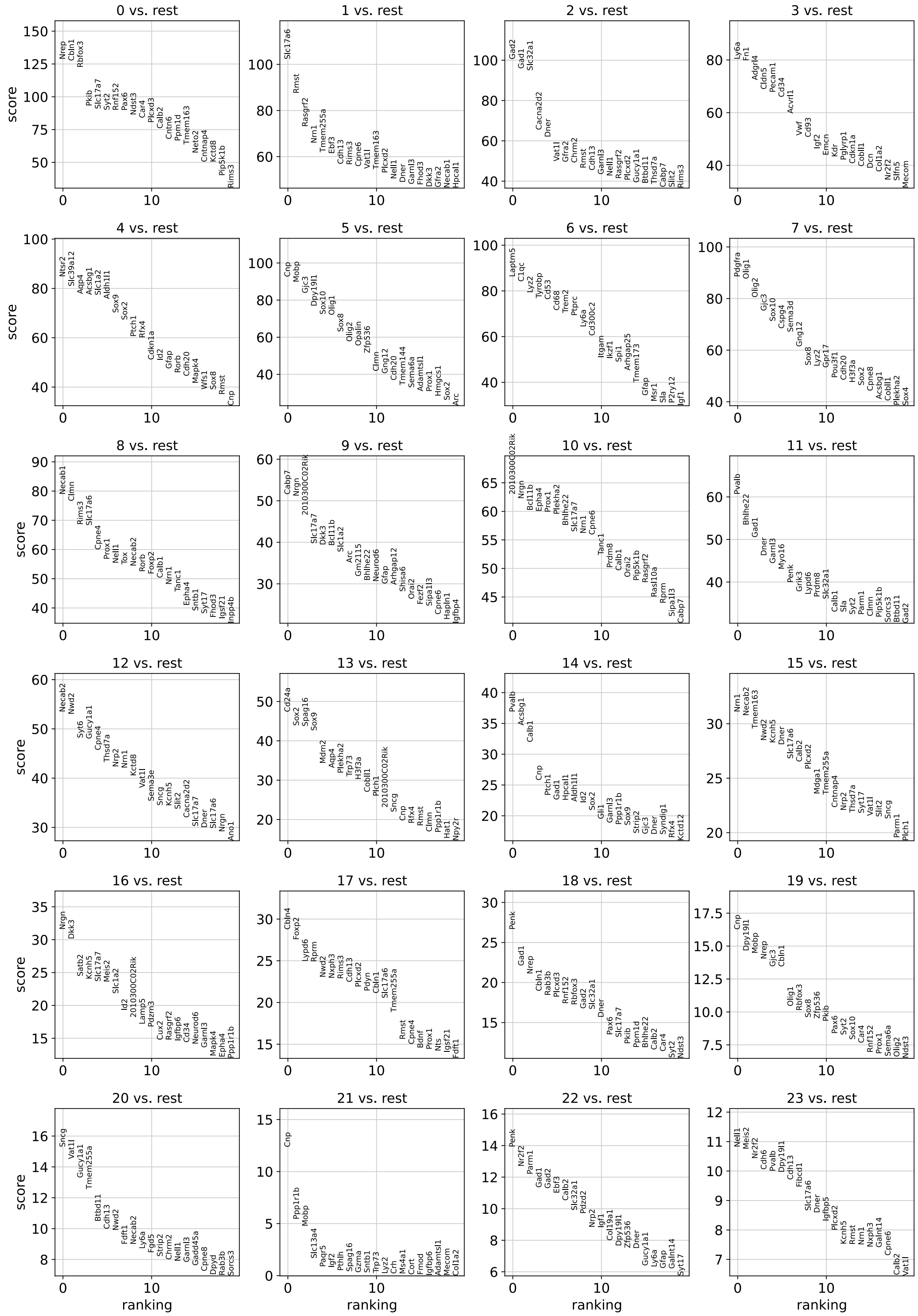

Supplemental Figure 4 - Differentially expressed marker genes for each cluster identified within all tumors (n=4).

Four plots show top differentially expressed genes for each cluster compared to all other clusters within the same sample. Four samples are shown, including Nestin^{TVA}; p53$^{FL/FL}$; H3f3a$^{LSL\text{-}K27M\text{-}Tag}$; Atm$^{FL/+}$(nPHA$^{FL/+}$) without irradiation; Nestin^{TVA}; p53$^{FL/FL}$; H3f3a$^{LSL\text{-}K27M\text{-}Tag}$; Atm$^{FL/+}$ (nPHA$^{FL/+}$) status post 10 Gy x 3 focal brain irradiation; Nestin^{TVA}; p53$^{FL/FL}$; H3f3a$^{LSL\text{-}K27M\text{-}Tag}$; Atm$^{FL/+}$ (nPHA$^{FL/FL}$) without irradiation; and Nestin^{TVA}; p53$^{FL/FL}$; H3f3a$^{LSL\text{-}K27M\text{-}Tag}$; Atm$^{FL/+}$ (nPHA$^{FL/FL}$) status post 10 Gy x 3 focal brain irradiation. Score represents relative fold change and significance of increased expression compared to all other groups.

Supplemental Figure 5

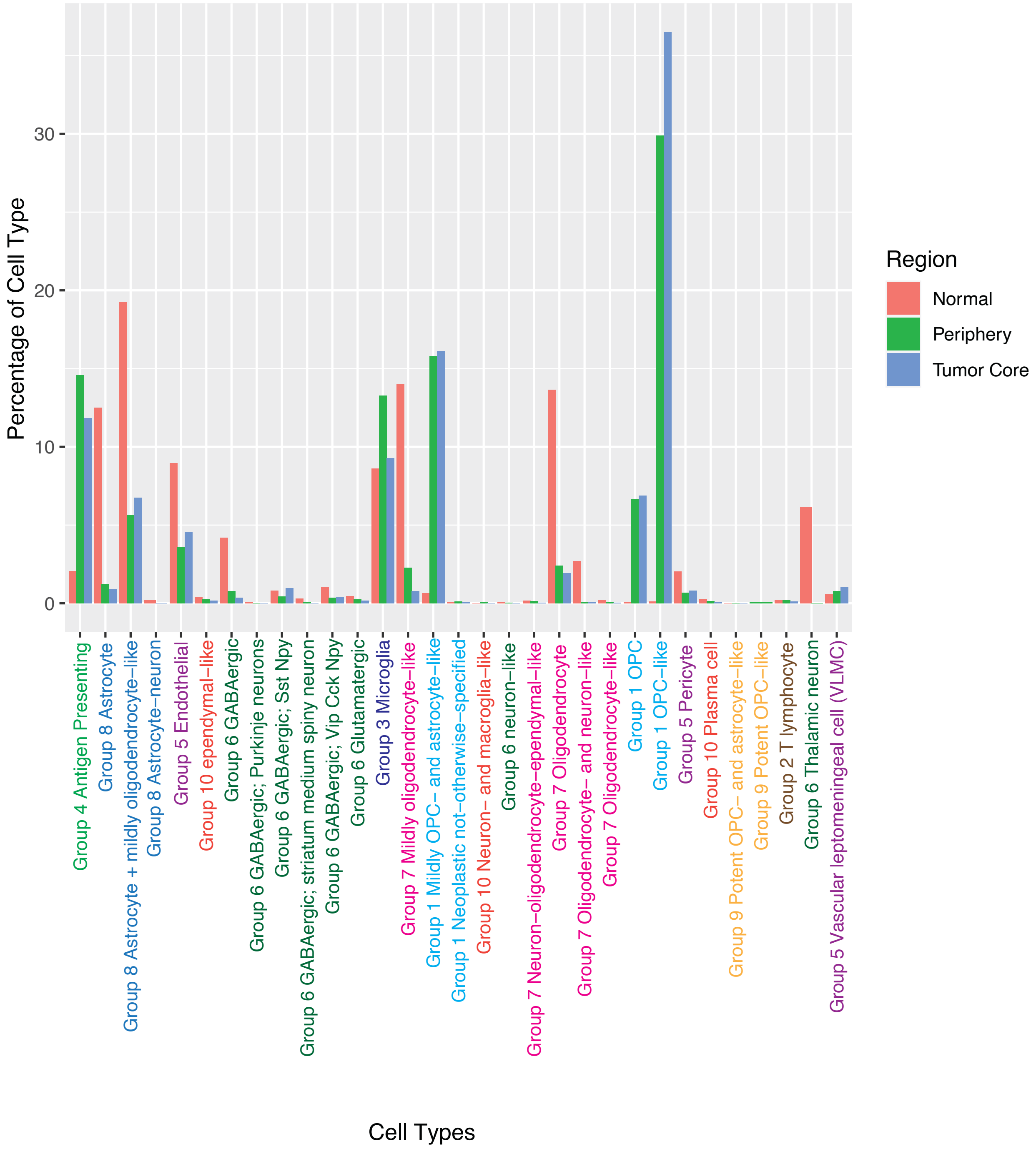

Supplemental Figure 5 – Collapsed individual cell clustering into 10 archetypal cell types.

Tumor core, periphery, and normal tissues were delineated as shown in Figure 3C. Bar graph shows percentage of cells of each type within each region. Grouping of UMAP clusters into 10 archetypal cell types is indicated. Cells are from unirradiated tumor-bearing nPHA$^{FL/FL}$ mouse brain shown in Supplemental Figure 3. All 4 samples were grouped utilizing the above described 10 archetypal cell types.

Supplemental Figure 6

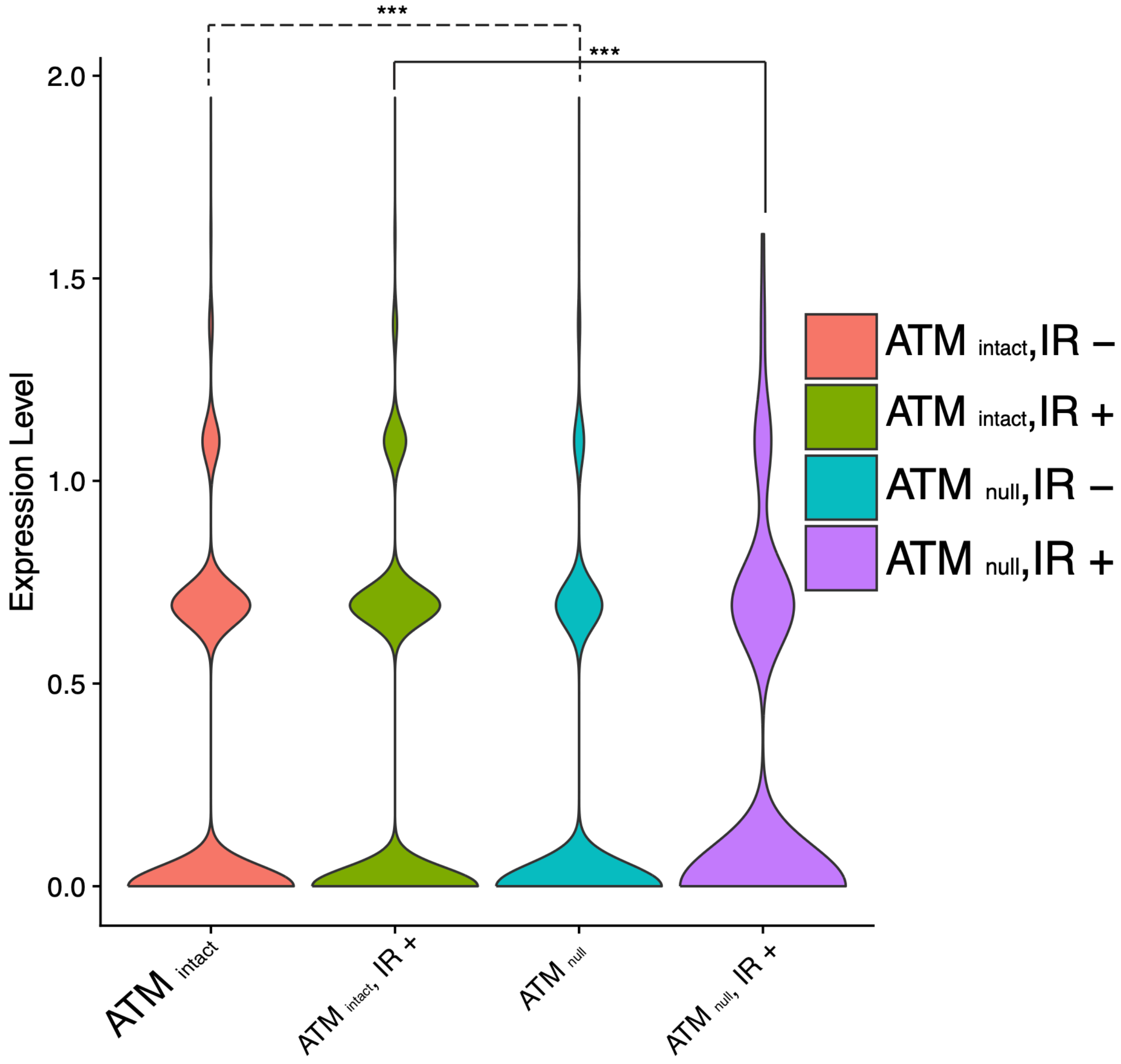

Supplemental Figure 6 - Violin plot with expression of Atm within Atm intact and Atm null tumors.
Violin plot showing ATM expression within each tumor genotype. ATM intact (n=1) was compared to ATM null (n=1) shows a statistically significant difference in ATM expression. ATM intact with irradiation (IR+, n=1) shows a statistically significant difference when compared to ATM null with irradiation (IR+, n=1) in ATM expression. Wilcoxon test was used to perform statistical analysis.

Supplemental Figure 7

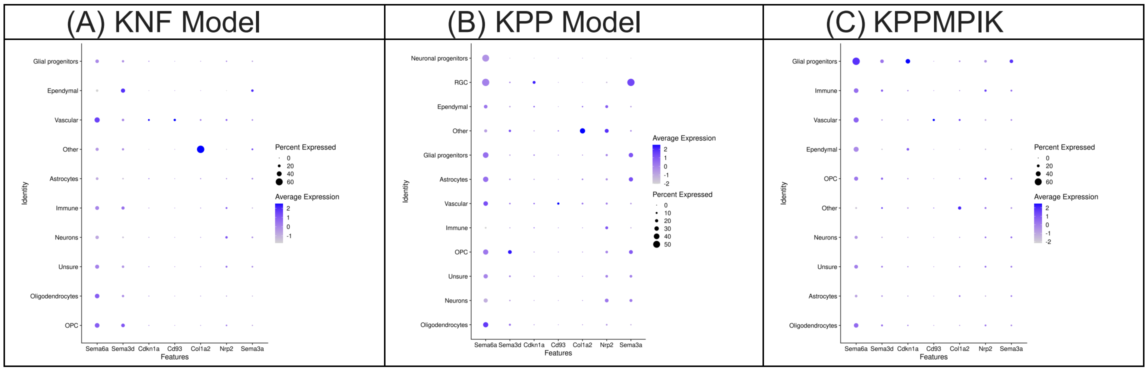

Supplemental Figure 7 - snRNA sequencing data from murine models to assess Semaphorin and p21 pathways

(A) Dot plot showing expression of H3.3^{K27M}, NF1^{LOF}, FGFR1^{N457K} (KNF model) of Sem6a, Sema3d, Cdkn1a, CD93, Col1a2, Nrp2, and Sema3a based on the consensus class annotation in the public dataset.

(B) Dot plot showing expression of H3.3^{K27M}, NF1^{LOF}, FGFR1^{N457K} (KPP model) of Sem6a, Sema3d, Cdkn1a, CD93, Col1a2, Nrp2, and Sema3a based on the consensus class annotation in the public dataset.

(C) Dot plot showing expression of H3.3^{K27M}, PPM1D$^{\Delta C}$, PIK3CA^{E545K} (KPPMPIK) of Sem6a, Sema3d, Cdkn1a, CD93, Col1a2, Nrp2, and Sema3a based on the consensus class annotation in the public dataset.

This dot plot was generated using the public dataset listed in this manuscript "McNicholas M, De Cola A, Bashardanesh Z, Foss A, Lloyd CB, Hébert S, Faury D, Andrade AF, Jabado N, Kleinman CL, Pathania M. A Compendium of Syngeneic, TransplanSupplemental Table Pediatric High-Grade Glioma Models Reveals Subtype-Specific Therapeutic Vulnerabilities. Cancer Discov. 2023 Jul 7;13(7):1592-1615. doi: 10.1158/2159-8290.CD-23-0004. PMID: 37011011; PMCID: PMC10326601."

Supplemental Figure 8

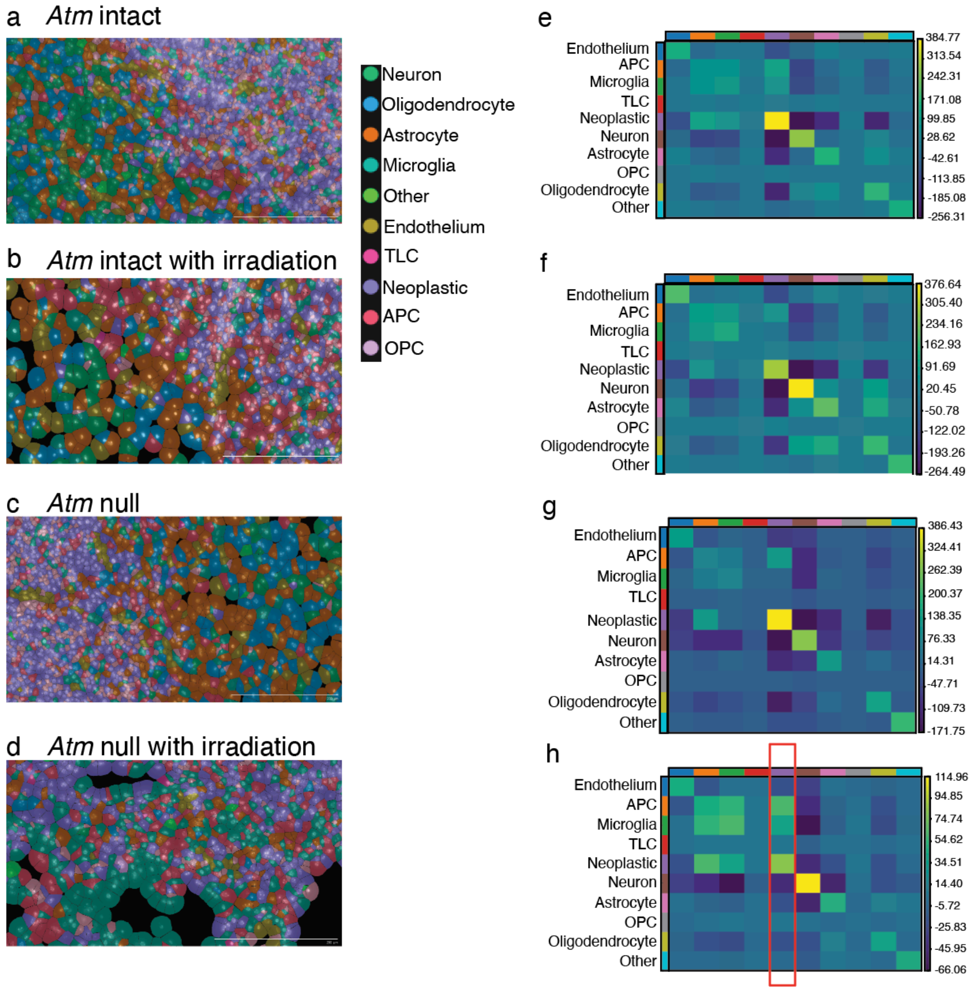

Supplemental Figure 8 - Neighborhood analysis of primary mouse DMGs comparing ATM tumoral loss and/or focal irradiation

(A) Representative image of *Atm*-intact (nPHAFL/+) tumor highlighting individual cell types identified by ISS at the border of normal brain and tumor.

(B) Representative image of *Atm*-intact (nPHAFL/+) tumor with irradiation highlighting individual cell types at the border of normal brain and tumor.

(C) Representative image of *Atm*-null (nPHAFL/FL) tumor highlighting individual cell types.

(D) Representative image of *Atm*-null (nPHAFL/FL) tumor with irradiation

(E) Neighborhood enrichment analysis of Atm-intact (nPHA FL/+) tumor showing proximity of various cell types in relationship to neoplastic cells.

(F) Neighborhood enrichment analysis of *Atm*-intact (nPHA FL/+) tumor with irradiation.

(G) Neighborhood enrichment analysis of *Atm*-null (nPHAFL/FL) tumor.

(H) Neighborhood enrichment analysis of *Atm*-null (nPHAFL/FL) tumor with irradiation showing proximity of various cell types in relationship to neoplastic cells. Red box indicates presence of antigen-presenting cells and microglia in relationship to neoplastic cells.

Supplemental Figure 9

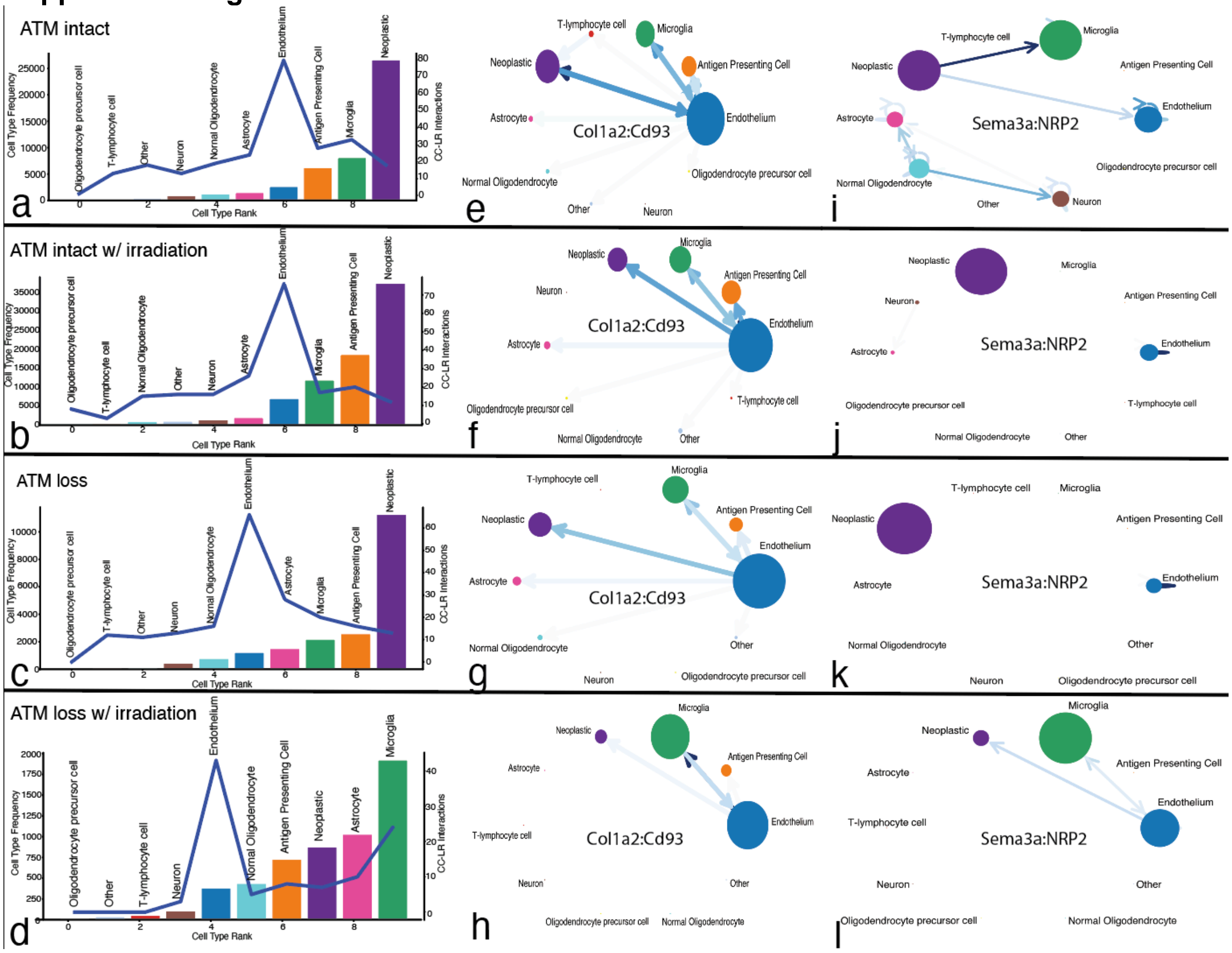

Supplemental Figure 9 - Spatial Cell Interactions within tumor environment of primary mouse DMGs with tumoral Atm loss with/without focal irradiation

(A-D) Bar graph demonstrating number of interactions with cells and ligand receptors of each cell type when compared to frequency of cells.

(E-H) Col1a2:Cd93 interaction amongst all cell types between *Atm* null (nPHAFL/FL) and *Atm* intact (nPHAFL/+). P-value < 0.05.

(I-L) Sema3a:NRP2 interaction amongst all cell types between neoplastic cells and endothelium between *Atm* null (nPHAFL/FL) and *Atm* intact (nPHAFL/+). P-value < 0.05.

Cell:Cell and Cell:Ligand interactions were conducted on tumor core and periphery. All unlabeled cells were removed for analysis.

Supplemental Figure 10

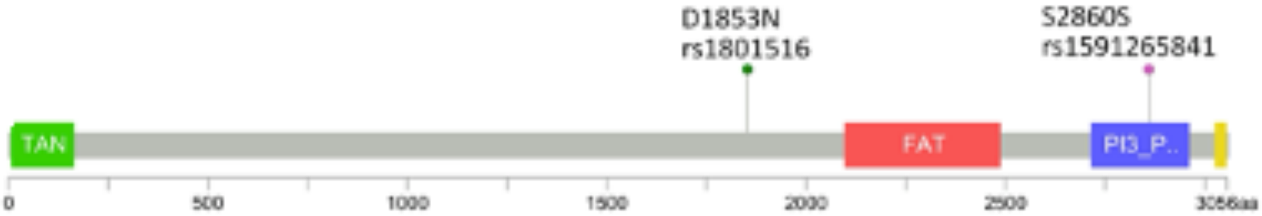

TAN: Tel1/ATM N-terminal motif
FAT: FRAP-ATM-TRRAP domain
PI3K_P:PI3K catalytic domain

Supplemental Figure 10 - ATM variants from RNA sequencing data using the GATK for patient derived cell line SF8628
RNA sequencing analysis of SF8628 does not show any pathologic ATM mutations. A missense and synonymous variant was identified on Chromosome 11.

Supplemental Figure 11

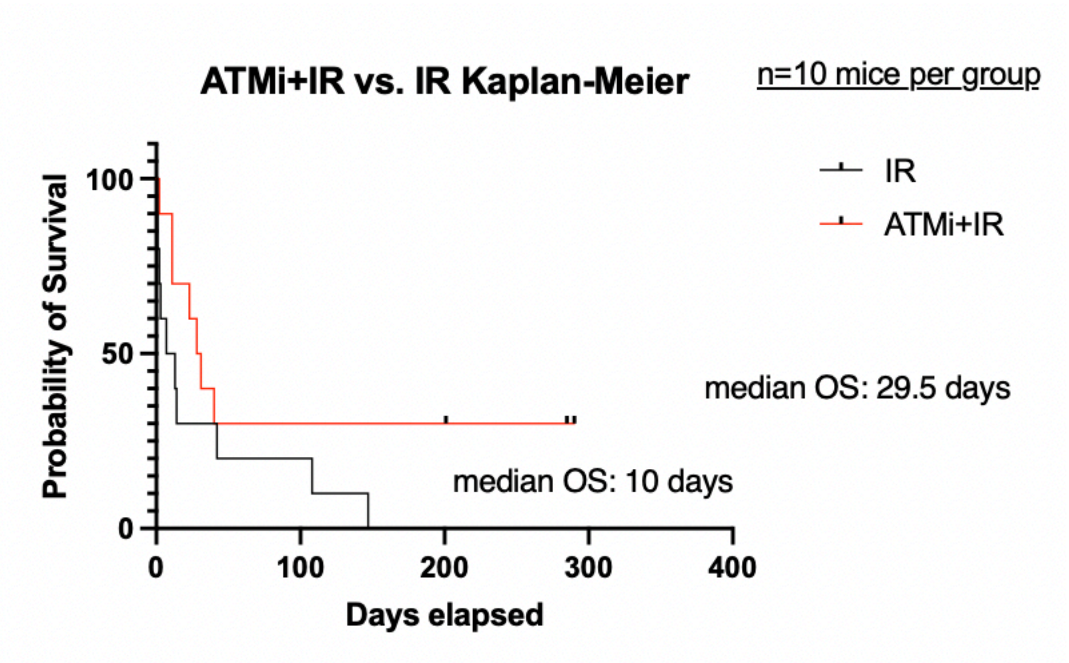

Supplemental Figure 11 - Survival curve of ATM intact genetically engineered murine glioma model with ATM inhibitor +/- irradiation
A Kaplan meier survival curve in a Nestin^{TVA} p53$^{FL/FL}$ model shows extended survival with a p-value = 0.1 when treated with AZD1390 plus irradiation compared to irradiation alone use a log-rank test.

Supplemental Figure 12

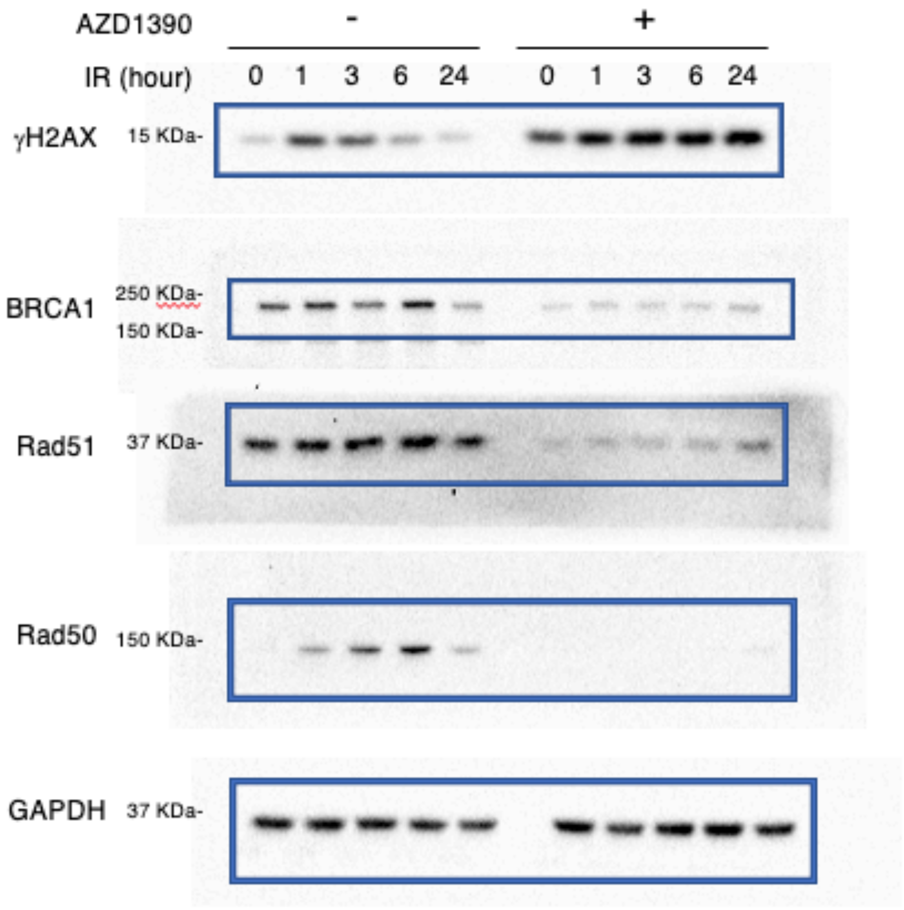

Supplemental Figure 12 - Raw images for western blot for DNA damage signaling in patient derived cell line, SF8628

Raw western blot images of SF8628 treated with and without AZD1390 and irradiation. Protein lysates were acquired over a time course following irradiation for γH2AX, BRCA1, Rad51, Rad50, and GAPDH. The molecular weights for the proteins are listed along with the blot.

Supplemental Figure 13

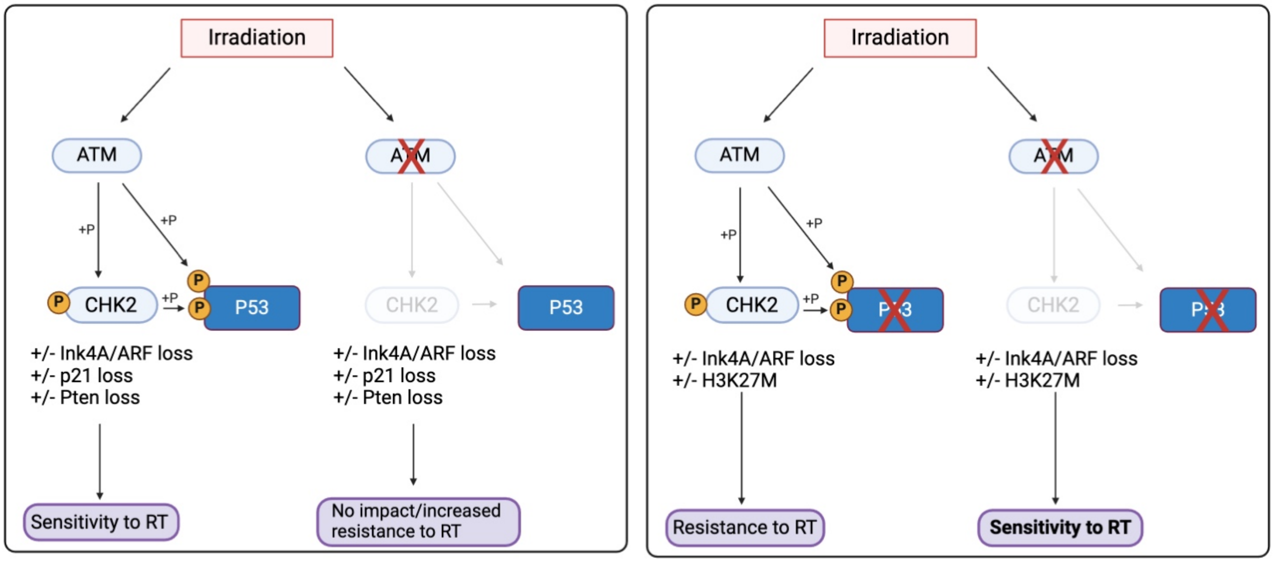

Supplemental Figure 13 - Concurrent ATM and p53 loss in GEMMs drive sensitivity to radiation therapy
Schematic of ATM acting as a "switch" that can radiosensitize cells in the context of p53 loss. Survival experiments described in this paper where the p53[25,26] allele was genetically engineered in mouse DMGs suggest that the increased efficacy of radiation therapy is not a result of p53's transactivation function.
Created in BioRender. Maingi, S. (2025) https://BioRender.com/r77l408

Supplemental Table 1 – Panel of 298 mouse brain and DMG transcripts targeted by in situ sequencing. (See separate Excel File).

Supplemental Table 2 – Collapsed individual cell clustering into 10 archetypal cell types.

Cell Type Identified by Xenium platform	Collapsed Cell Clustering
Mildly OPC- and astrocyte-like<br>Neoplastic not-otherwise specified<br>OPC<br>OPC-like	Neoplastic (1)
T-lymphocyte	T-Lymphocyte Cell (2)
Microglia	Microglia (3)
Antigen Presenting Cell	Antigen Presenting Cell (4)
Endothelial	Endothelium (5)
GABAergic<br>Purkinje Neurons<br>GABAergic; Sst Npy<br>GABAergic; stratum medium spiny neuron<br>GABAergic; Vip Cck Npy<br>Glutamatergic<br>Neuron-like<br>Thalamic Neuron	Neuron (6)
Mildly Oligodendrocyte-like<br>Neuron-oligodendrocyte-ependymal-like<br>Oligodendrocyte<br>Oligodendrocyte-and neuron-like<br>Oligodendrocyte-like	Normal Oligodendrocyte (7)
Astrocyte<br>Astrocyte + mildly oligodendrocyte like<br>Astrocyte neuron	Normal Astrocyte (8)
Potent OPC-and Astrocyte like<br>Potent OPC-like	Normal Oligodendrocyte precursor cell (9)
Ependymal – like<br>Plasma Cell	Other: Ependymal cells, Plasma cell etc. (10)

Supplemental Table 3 - Top differentially expressed genes of *Atm* intact (FL/+) with and without irradiation.

$Atm^{FL/+}$ with and without irradiation

Differentially Expressed Genes	p_val	avg_log2FC	pct.1	pct.2	p_val_adj
Cd24a	0	0.45465633	0.535	0.371	0
Cdkn1a	0	0.82625756	0.305	0.156	0
Cntn6	0	-0.9533859	0.074	0.217	0
Col19a1	0	-0.6388915	0.414	0.614	0
Cpne8	0	0.65663785	0.701	0.535	0
Dpy19l1	0	-0.6679676	0.715	0.862	0
Gjc3	0	0.38412926	0.966	0.939	0
Gng12	0	-0.2903972	0.957	0.978	0
Gpr17	0	-1.0042095	0.675	0.903	0
Igfbp4	0	0.84272815	0.599	0.399	0
Mobp	0	-1.0655137	0.061	0.181	0
Neto2	0	0.59804994	0.75	0.616	0
Ntsr2	0	-0.8856547	0.11	0.251	0
Pde7b	0	0.64834639	0.482	0.33	0
Pou3f1	0	-1.7321007	0.431	0.863	0
Pten	0	0.43419965	0.942	0.887	0
Rprm	0	-0.5341461	0.458	0.61	0
Sema3a	0	0.81370153	0.34	0.185	0
Sema3d	0	1.13596382	0.823	0.553	0
Sox2	0	-0.330949	0.967	0.986	0
Sox8	0	-0.5154122	0.781	0.894	0
Sox9	0	-1.1872076	0.186	0.442	0
Syt6	0	0.86444409	0.279	0.125	0
Meis2	1.25E-283	0.5602549	0.544	0.408	3.73E-281
Zfp536	1.47E-271	0.61154868	0.413	0.271	4.39E-269
Arc	1.68E-271	0.88155626	0.209	0.095	5.00E-269
Tmem132d	1.05E-232	-0.4974553	0.368	0.496	3.13E-230
Dner	1.17E-226	-0.3220224	0.756	0.838	3.50E-224
Rab3b	4.26E-224	0.53254171	0.462	0.335	1.27E-221
Fdft1	3.24E-220	-0.4163337	0.486	0.608	9.65E-218
Cnp	1.80E-209	0.30838304	0.78	0.692	5.38E-207
Aldh1l1	7.73E-199	0.8521914	0.177	0.086	2.30E-196
Mcm2	1.32E-165	0.41357323	0.621	0.53	3.94E-163

Tox	9.79E-161	-0.4969958	0.158	0.25	2.92E-158
Top2a	1.10E-159	0.28204286	0.889	0.836	3.28E-157
Arhgap25	5.41E-144	-0.5362523	0.055	0.114	1.61E-141
Gucy1a1	2.97E-139	0.51777822	0.239	0.151	8.85E-137
Rorb	8.12E-131	0.40191759	0.5	0.407	2.42E-128
Cdh13	1.10E-125	0.33294844	0.627	0.547	3.28E-123
Fos	3.16E-123	0.57973269	0.237	0.156	9.43E-121
Gfap	7.85E-122	-0.3460217	0.283	0.381	2.34E-119
Nrep	5.20E-121	-0.2983228	0.502	0.596	1.55E-118
Thsd7a	3.60E-119	0.44123699	0.373	0.284	1.07E-116
2010300C02Rik	1.62E-111	-0.4515898	0.085	0.145	4.83E-109
Lyz2	1.82E-111	0.49140701	0.206	0.13	5.42E-109
Dkk3	1.08E-109	0.46464082	0.231	0.153	3.21E-107
Plekha2	4.67E-104	0.29636309	0.629	0.554	1.39E-101
Tanc1	2.84E-101	0.27519069	0.663	0.592	8.45E-99
Calb2	1.90E-94	-0.9437316	0.06	0.107	5.65E-92
Plcxd2	2.53E-88	-0.4390091	0.07	0.119	7.55E-86
Prss35	4.92E-85	0.48382686	0.12	0.069	1.47E-82
Kcnh5	1.69E-78	0.36233524	0.357	0.285	5.02E-76
Arhgef28	2.13E-78	0.40198553	0.134	0.081	6.34E-76
Kctd12	8.34E-77	0.31845716	0.372	0.298	2.49E-74
Rims3	6.20E-76	-0.4395223	0.105	0.158	1.85E-73
Pdzrn3	2.95E-74	0.49602445	0.136	0.085	8.78E-72
Prdm8	3.32E-74	-0.3600763	0.082	0.131	9.90E-72
Lypd6	1.63E-63	-0.3019273	0.289	0.354	4.85E-61
Igsf21	9.68E-63	0.28647183	0.395	0.33	2.88E-60
Hpcal1	1.41E-60	-0.3192566	0.273	0.336	4.19E-58
Fn1	6.35E-54	0.30947881	0.215	0.161	1.89E-51
Foxp2	2.29E-51	-0.2855498	0.201	0.255	6.82E-49
Bcl11b	7.91E-49	0.32651332	0.103	0.066	2.36E-46
Plch1	2.90E-48	0.31871345	0.129	0.088	8.66E-46
Laptm5	1.40E-43	-0.3700916	0.094	0.131	4.18E-41
Zfpm2	4.54E-39	0.26389695	0.249	0.203	1.35E-36
Cabp7	4.65E-39	0.30337275	0.131	0.094	1.38E-36
Igfbp5	1.37E-33	0.38005264	0.195	0.156	4.07E-31
C1qc	2.01E-33	-0.3428421	0.138	0.174	5.99E-31

Supplemental Table 4 –Top differentially expressed genes of *Atm* null (FL/FL) with and without irradiation.

$Atm^{FL/FL}$ with and without irradiation DEGs

Differentially Expressed Genes	P-value	Average Log_2fold change	pct.1	pct.2	p-value adjusted
Ly6a	0	2.98215354	0.859	0.172	0
Lyz2	0	4.23344401	0.919	0.107	0
Igf1	3.94E-222	1.80694116	0.267	0.019	1.18E-219
Ndst3	1.30E-199	1.85249795	0.509	0.104	3.87E-197
Cd44	2.51E-197	2.13172941	0.407	0.065	7.49E-195
Acsbg1	5.78E-183	1.43591491	0.946	0.618	1.72E-180
Pde11a	1.97E-154	1.48705741	0.196	0.015	5.88E-152
Slfn5	1.22E-144	1.60425171	0.284	0.041	3.64E-142
Hmgcs1	2.43E-132	-1.5009082	0.589	0.857	7.24E-130
C1qc	5.77E-113	1.57155156	0.65	0.275	1.72E-110
Ccn2	2.64E-110	1.5840533	0.208	0.028	7.88E-108
Plch1	4.18E-108	1.98761893	0.314	0.07	1.25E-105
H3f3a	2.70E-102	-0.482292	0.996	0.999	8.04E-100
Ptprc	1.74E-95	1.5100652	0.179	0.024	5.17E-93
Laptm5	1.08E-93	1.41268304	0.489	0.173	3.23E-91
Pdgfra	6.05E-92	-0.4518301	0.99	1	1.80E-89
Cd68	4.20E-78	1.4727131	0.341	0.104	1.25E-75
Cspg4	5.05E-78	-0.6589742	0.898	0.976	1.51E-75
Fdft1	1.09E-75	-1.4165217	0.347	0.66	3.25E-73
Olig2	8.61E-65	-0.4715824	0.976	0.997	2.56E-62
Top2a	4.92E-64	-1.7486486	0.186	0.521	1.47E-61
Tyrobp	7.61E-63	1.18999988	0.336	0.114	2.27E-60
Cdh4	1.30E-60	-1.4784587	0.181	0.496	3.86E-58
Pten	1.57E-58	0.5696656	0.967	0.879	4.68E-56
Cd53	2.70E-56	1.19715954	0.243	0.07	8.04E-54
Cabp7	5.69E-55	1.08922467	0.346	0.13	1.69E-52
Gng12	6.32E-54	-0.5100133	0.945	0.975	1.88E-51
Tmem132d	7.66E-49	-0.9016209	0.501	0.724	2.28E-46
Cd24a	1.40E-45	1.06924757	0.528	0.295	4.17E-43
Arhgef28	1.35E-44	0.96203677	0.347	0.146	4.02E-42
Col1a2	6.15E-44	1.23832124	0.141	0.032	1.83E-41
Sdk2	8.38E-43	0.88307863	0.129	0.028	2.50E-40
Sox2	1.27E-42	-0.4453391	0.917	0.964	3.80E-40

Pou3f1	6.24E-40	0.77351178	0.707	0.505	1.86E-37
Slc1a2	1.22E-39	0.37697851	0.99	0.97	3.62E-37
Col6a1	6.08E-38	1.09447317	0.101	0.02	1.81E-35
Tmem144	8.59E-38	0.91615103	0.397	0.196	2.56E-35
Parm1	2.67E-36	1.10065967	0.249	0.096	7.95E-34
Slit2	2.97E-34	-0.862754	0.306	0.542	8.86E-32
Dner	2.81E-33	-0.3945525	0.903	0.945	8.38E-31
Sox4	5.49E-33	-0.5721428	0.69	0.82	1.63E-30
Gfap	1.25E-31	0.60138056	0.836	0.692	3.72E-29
Fn1	1.56E-30	1.00256142	0.269	0.118	4.65E-28
Meis2	2.67E-29	-0.5883184	0.514	0.694	7.95E-27
Igfbp4	1.20E-27	0.78252666	0.63	0.464	3.57E-25
Gadd45a	1.35E-27	0.95178348	0.364	0.195	4.01E-25
Bhlhe40	7.47E-26	0.77620985	0.428	0.252	2.23E-23
Angpt1	6.51E-24	0.76308213	0.312	0.163	1.94E-21
Gm2115	6.56E-24	0.74811419	0.182	0.073	1.95E-21
Cd300c2	7.19E-23	0.72751193	0.168	0.066	2.14E-20
Lypd6	9.88E-23	0.77382067	0.35	0.198	2.94E-20
Necab2	6.97E-22	-0.7398512	0.334	0.503	2.08E-19
Hpcal1	7.67E-22	0.61519381	0.454	0.284	2.29E-19
Gucy1a1	8.65E-22	-0.7476419	0.297	0.474	2.58E-19
Pdzd2	9.60E-22	0.61992912	0.501	0.335	2.86E-19
Trp53	2.11E-21	0.70218841	0.11	0.035	6.30E-19
Gjc3	4.19E-21	0.29941251	0.99	0.986	1.25E-18
Cobll1	1.26E-20	0.47969117	0.71	0.556	3.74E-18
Acta2	1.89E-20	0.82247983	0.199	0.09	5.62E-18
Aqp4	4.46E-20	0.74658106	0.448	0.292	1.33E-17
Wfs1	1.35E-19	0.71863796	0.205	0.096	4.03E-17
2010300C02Rik	2.00E-19	-0.8845026	0.121	0.275	5.97E-17
Cntnap5b	3.56E-19	-0.93947	0.084	0.229	1.06E-16
Trem2	6.39E-19	0.66197924	0.193	0.089	1.90E-16
Rab3b	9.52E-19	-0.6141688	0.31	0.477	2.84E-16
Dpyd	6.21E-18	0.51193471	0.596	0.449	1.85E-15
Tox	1.80E-17	-0.6745374	0.193	0.35	5.35E-15
Sema6a	2.21E-17	-0.4048593	0.777	0.844	6.59E-15
Slc44a5	5.01E-16	-0.7426964	0.165	0.31	1.49E-13
Gfra2	5.07E-16	0.73275394	0.299	0.18	1.51E-13
Slc39a12	1.45E-15	0.63998648	0.131	0.055	4.32E-13
Spi1	3.26E-15	0.50595543	0.102	0.038	9.72E-13

Supplemental Table 5 – Top Cell Ligand receptors with a p-value < 0.05 for all tumors.

Atm intact

	n_spots	n_spots_sig	n_spots_sig_pval	n_cci_sig_celltype	n-spot_cci_celltype	n-spot_cci_sig_celltype
Col1a2_Cd93	23723	2694	5124	14	14021	6686
Col1a1_Cd93 1	10095	1855	4283	10	8528	5338
Fn1_Cd44	16090	1839	3039	16	13541	7294
Col1a2_Cd44	8273	759	1273	17	3847	2469
Spp1_Cd44	6069	732	1269	14	4585	3433
Col1a1_Cd44	3770	592	1128	14	2863	2024
Sema3a_Nrp2	9926	501	1055	15	1840	442
Nts_Ntsr2	5486	440	863	25	1845	678
Ccn2_Itgam	6480	437	893	10	1364	1169

Atm intact with irradiation

	n_spots	n_spots_sig	n_spots_sig_pval	n_cci_sig_celltype	n-spot_cci_celltype	n-spot_cci_sig_celltype
Fn1_Cd44	40314	3450	6849	15	30215	22395
Col1a2_Cd93	41079	2149	3909	9	18043	14757
Col1a1_Cd93	21745	1729	3659	8	13885	11506
Col1a2_Cd44	20598	1468	2270	10	13946	11471
Spp1_Cd44	19217	1267	2254	13	12441	9660
Col1a1_Cd44	11956	1139	1803	10	10470	8721
Ccn2_Itgam	16744	801	1542	10	3629	3134
Sema3a_Nrp2	27072	669	2032	2	3381	1172
Nts_Ntsr2	7012	629	1407	36	2629	1435

Atm null

	n_spots	n_spots_sig	n_spots_sig_pval	n_cci_sig_celltype	n-spot_cci_celltype	n-spot_cci_sig_celltype
Fn1_Cd44	4784	783	1183	12	4999	4292
Col1a2_Cd93	4426	447	736	8	3291	3123
Col1a1_Cd93	2580	426	633	8	2987	2811
Nts_Ntsr2	2199	326	445	34	2958	1776
Spp1_Cd44	2371	289	489	10	1738	1402
Col1a2_Cd44	2425	250	467	12	1430	1084
Sema3a_Nrp2	4543	239	346	3	1009	562
Ccn2_Itgam	3315	209	447	7	1045	866
Col1a1_Cd44	1427	208	378	12	1208	1086

Atm null with irradiation

	n_spots	n_spots_sig	n_spots_sig_pval	n_cci_sig_celltype	n-spot_cci_celltype	n-spot_cci_sig_celltype
Fn1_Cd44	3572	276	663	8	2006	1521
Col1a2_Cd93	1807	139	298	6	852	763
Col1a2_Cd44	2432	133	276	7	887	678
Ccn2_Itgam	2142	110	232	6	575	465
Spp1_Cd44	2846	101	302	7	671	433
Nts_Ntsr2	1062	68	213	10	311	195
Col1a1_Cd93	671	64	164	6	286	255
Col1a1_Cd44	832	47	139	5	266	148
Sema3a_Nrp2	1694	42	144	4	191	89

Supplemental Table 6 - ATM variant in patient derived cell line SF8628.

Location (hg38)	Symbol	REF	ALT	dbSNP ID	Consequence	Codon change	Protein Change
Chr11:108304735	ATM	G	A	rs1801516	Missense	Gat/Aat	D1853N
Chr11:108345904	ATM	T	C	rs1591265841	Synonymous	tcT/tcC	S2860S

Supplemental Table 7 – List of Abbreviations

Name	Abbreviation
Diffuse Midline Glioma	DMG
Ataxia Telangiectasia mutated	Atm
Genetically engineered mouse model	GEMM
Cyclin dependent kinase inhibitor 1A	Cdkn1a
Uniform Manifold Approximation and Projection	UMAP
Semaphorin 3a	Sema3a
Semaphorin 3d	Sema3d
Semaphorin 6a	Sema6a
Platelet derived growth factor receptor A	Pdgfra
Oligodendrocyte transcription factor 1	Olig1
Oligodendrocyte transcription factor 2	Olig2
SRY-box transcription factor 1	Sox1
SRY-box transcription factor 2	Sox2
SRY-box transcription factor 4	Sox4
SRY-box transcription factor 8	Sox8
SRY-box transcription factor 9	Sox9
Glutamate decarboxylase 1	Gad1
Glutamate decarboxylase 2	Gad2
Purigenic receptor P2Y12	P2ry12
Lysozyme 2	Lyz2

Complement C1q A	C1qa
Fibronectin 1	Fn1
Adhesion G Protein Coupled Receptor 14	Adgr14
Collagen type 1 alpha 2 chain	Co11a2
Neuropilin 2	NRP2
Phosphatase and tensin homolog	Pten
Terminal deoxynucleotidyl transferase dUTP nick end labeling	TUNEL
Replication competent avian sarcoma/tumor virus receptor A	RCAS/TVA
Institutional Animal Care and Use Committee	IACUC
4’,6-diamidine-2-phenylindole	DAPI
Formalin fixed paraffin embedded	FFPE
In situ sequencing	ISS
Short Tandem Repeats	STR

Supplemental Table 8 - Key resources table

REAGENT or RESOURCE	SOURCE	IDENTIFIER
**Antibodies**		
Rabbit polyclonal HA-probe	Santa Cruz Biotechnology	Cat# sc-805 RRID: AB_631618
Mouse monoclonal Ser1981 phosphorylated ATM	MilliporeSigma	Cat# 05740 RRID: AB_2062670
Rabbit anti-mouse Ser824 phosphorylated Kap1	ThermoFisher	Cat# A300-767A RRID:AB_2779445
Rabbit polyclonal IgG p21	Santa Cruz Biotechnology	Cat# sc-471 RRID:AB_383248
Mouse TotalKap1	Bethyl Laboratories	Cat#A300-775A
Mouse γH2AX	Millipore	Cat# 05-636
**Chemicals, peptides, and recombinant proteins**		
D-luciferin, potassium salt	Gold Biotechnology	Cat# LUCK-1G
**Critical commercial assays**		
PicoPure DNA extraction kit	ThermoFisher	Cat# KIT0103
**Cell Media and supplements**		
Dulbecco's Modified Eagle's medium	ThermoFisher	Cat# 11965092
10% Fetal Bovine Serum	ThermoFisher	Cat# A31604-02

Non-Essential Amino Acids	ThermoFisher	Cat#11140-050
**Experimental models: Cell lines**		
UMNSAH/DF-1 chicken fibroblast cells	ATCC	CRL-12203^{TM}
SF8628 Human Cell line DIPG H3.3-K27M	University of California San Francisco (UCSF)	Developed by Dr. Rintaro Hashizume at UCSF
**Experimental models: Organisms/strains**		
Mouse: *Nestin*^{TVA}: Tg(NES-TVA)J12Ech/J	The Jackson Laboratory	RRID:IMSR_JAX:003529
Mouse: H3f3a-LSL-K27M-Tag	Lab of Dr. Suzanne Baker	N/A
Mouse: LSL-p53-25,26	Lab of Dr. Laura Attardi	N/A
Mouse: p21-/-	The Jackson Laboratory	RRID:IMSR_JAX:016565
Mouse: *p53*^{fl}: B6.129P2-Trp53tm1Brn/J	The Jackson Laboratory	RRID:IMSR_JAX:008462
Mouse: *ATM*^{fl}: 129-Atmtm2.1Fwa/J	The Jackson Laboratory	RRID:IMSR_JAX:021444

Mouse: *Ink4a/Arf*^{fl}: Cdkn2atm1Rdp	Mouse Genome Informatics	MGI: 1857942
Athymic Mice (rnu/rnu genotype, BALB/c background)	Envigo	Code: 069
**Oligonucleotides**		
Primers for *p53*^{fl} alleles	Weidenhammer et al., 2023	N/A
Primers for *Ink4a/Arf*^{fl} alleles, see table S1	Weidenhammer et al., 2023	N/A
Atm recombined probe, 5′-ACACATGCATGCAGGCAGAGCATCCCT-3′	Weidenhammer et al., 2023	N/A
Atm-floxed probe, 5′-AGCTGTTACTTTTGCGTTTGGTGTGGCG-3′	Weidenhammer et al., 2023	N/A
p53 recombined probe, 5′-CTTGATATCGAATTCCTGCAGCCCGGG-3′	Weidenhammer et al., 2023	N/A
p53 floxed probe, 5′-ATGCTATACGAAGTTATCTGCAGCCCGG-3′	Weidenhammer et al., 2023	N/A

Ink4a/Arf recombined probe, 5′-CATTATACGAAGTTATGGCGCGCCC-3′	Weidenhammer et al., 2023	N/A
Ink4a/Arf floxed probe, 5′-CTCTGAAAACCTCCAGCGTATTCTGGTA-3′	Weidenhammer et al., 2023	N/A
**Recombinant DNA**		
Plasmid: RCAS-Cre	Barton KL, et al., 2013	N/A
Plasmid: RCAS-Luc	Laboratory of Oren Becher	N/A
Plasmid: RCAS-PDGFB	Barton KL, et al., 2013	N/A
**Software and algorithms**		
ImageJ	NIH	https://imagej.nih.gov/ij/
Prism 7	GraphPad Software Inc.	https://www.graphpad.com/scientific-software/prism/

QuantaSoft	Bio-rad	https://www.bio-rad.com/en-us/life-science/digital-pcr/qx200-droplet-digital-pcr-system/quantasoft-software-regulatory-edition?ID=1864011
Xenium Analyzer	10xGenomics	https://www.10xgenomics.com/instruments/xenium-analyzer
Seurat	Satija Lab	https://satijalab.org/seurat/
StLearn	Stlearn	https://stlearn.readthedocs.io/en/latest/
Squidpy	Squidpy	https://squidpy.readthedocs.io/en/stable/
STRING	STRING	https://string-db.org
HOMER Motif Analysis	HOMER	http://homer.ucsd.edu/homer
Trimmomatic	Trimmomatic	Version 0.36
Bismark Software	Bismark	Version 0.24.0
**Other**		
Biological Safety Cabinet	Thermo Fisher Scientific	Cat#13-261-222

IVIS Lumina III In Vivo Imaging System	PerkinElmer	Cat#CLS136334
CO_2 Incubators	Thermo Fisher Scientific	Cat#4110
EVOS M7000 Imaging System	Thermo Fisher Scientific	Cat#AMF7000
Hamilton Syringe	Hamilton	Cat#84851
Isoflurane Vaporizer	Kent Scientific	Cat#VetFlo-1205S
Oxygen Concentrator	Fisher Scientific	Cat#04-777-122
Sure-Seal Large Mouse/Rat Induction Chamber	World Precision Instruments	Cat#EZ-1785
Sterile Sleeves	VWR	Cat#414004-510
TUNEL DeadEnd Colorimetric System	Promega	Cat#G7360
AZD1390	Astrazeneca	N/A
Mycoplasma Detection Kit	InvivoGen	Cat#rep-mys-10
Short Tandem Repeat	Promega PowerPlex 16 HS System	Cat#DC2101

Materials and equipment

D-Luciferin Stock Solution

Reagent	Final concentration	Amount

D-Luciferin, Potassium Salt	n/a	1 g
Dulbecco's Phosphate Buffered Saline without Ca^{2+} and Mg^{2+}	1X	66.6 mL
**Total**	**15mg/mL**	**66.6 mL**

Store at -80°C; expires after 1 year

Alternatives: D-Luciferin Sodium Salt and L-Luciferin Potassium Salt can be substitute for D-Luciferin, Potassium Salt
