# Supplementary figures and images for: Disruption of ataxia telangiectasia–mutated kinase enhances radiation therapy efficacy in spatially directed diffuse midline glioma models
Source: J Clin Invest. 2025 Apr 17;135(12):e179395. doi: 10.1172/JCI179395 (PMC12165813; doi:10.1172/JCI179395)

# Raw Image Supplemental Figure 12

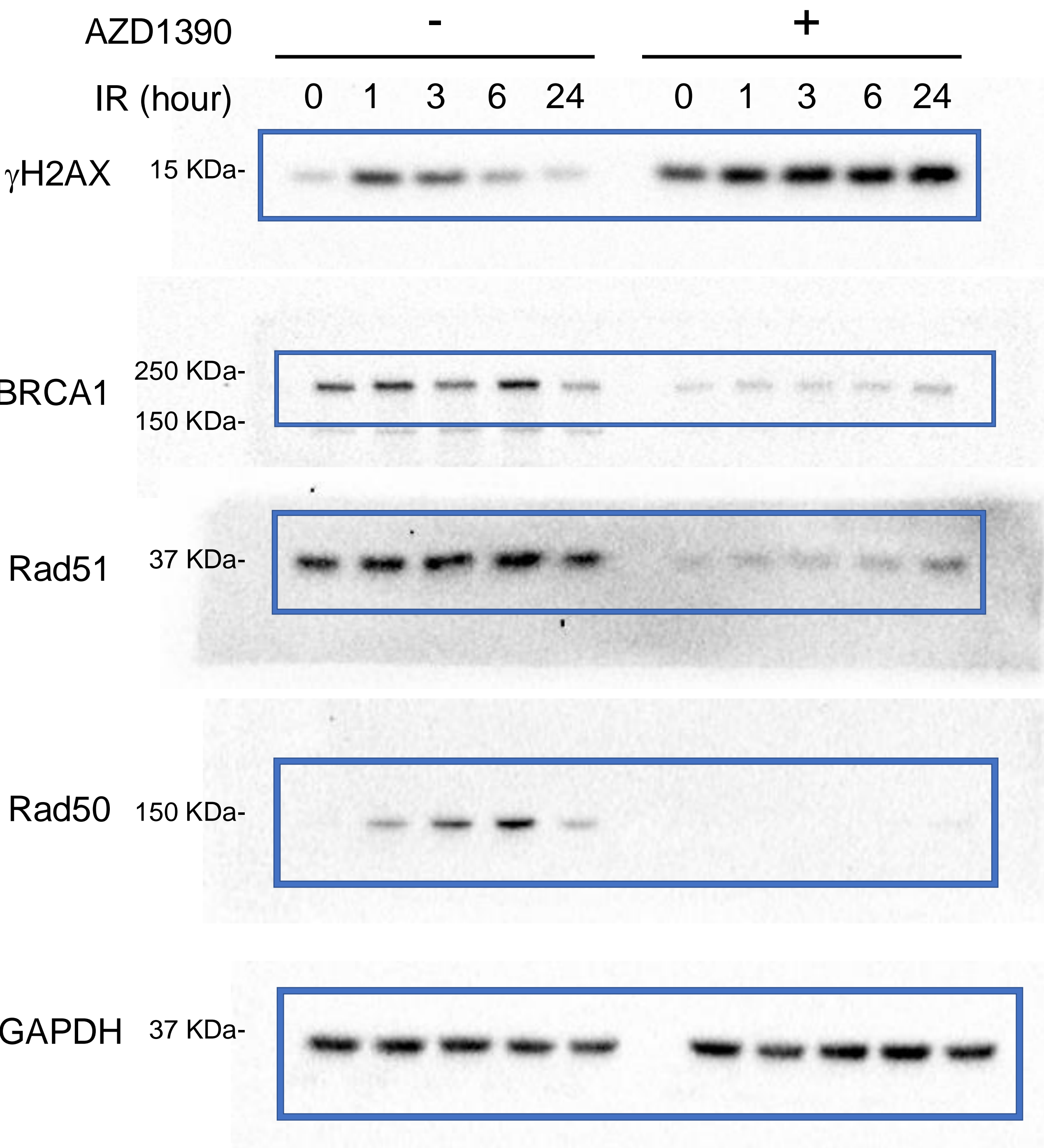

Supplement: Unedited blot and gel images [file jci-135-179395-s056.pdf]
